# Biocontrol of *Kosakonia radicincitans* and *Paraburkholderia phytofirmans* against Botrytis and Fusarium in tomato: role of potential inducing resistance and pathogen-specific responses

**DOI:** 10.3389/fpls.2026.1775113

**Published:** 2026-05-14

**Authors:** Mohamed Matared, Imke Hutter, Katja Burow, Philipp Franken

**Affiliations:** 1 INOQ GmbH, Schnega, Germany; 2 Institute of Microbiology, Friedrich Schiller University Jena, Jena, Germany; 3 Erfurt Research Centre for Horticultural Crops, University of Applied Sciences Erfurt, Erfurt, Germany; 4 Department of Vegetable and Aromatic Plant Insects, Agricultural Research Center, Plant Protection Research Institute, Sabahia Plant Protection Research Station, Alexandria, Egypt; 5 Occupational Safety Department, Biological Safety, Friedrich Schiller University Jena, Jena, Germany

**Keywords:** biocontrol, *Botrytis cinerea*, *Fusarium oxysporum*, inducing resistance, *Kosakonia* radicincitans, *Paraburkholderia phytofirmans*, PGPB, priming

## Abstract

**Introduction:**

Plant growth-promoting bacteria (PGPB) are increasingly recognized for their capacity to enhance plant growth and, in some cases, to induce systemic resistance against phytopathogens, though the extent and specificity of this protection remain strain- and pathosystem-dependent. This study investigates the biocontrol potential of two endophytic PGPB strains, *Kosakonia radicincitans* and *Paraburkholderia phytofirmans*, against the necrotrophic pathogen *Botrytis cinerea* and the soilborne hemibiotrophic pathogen *Fusarium oxysporum* f.sp. *lycopersici* in tomato plants (*Solanum lycopersicum* L.).

**Methods:**

We tested both strains for their antagonistic potential in dual culture and two-compartment Petri plates todetermine their direct antifungal capacity under controlled conditions. After this, tomato plants were pre-inoculated with each PGPB and challenged with *B. cinerea* or *F. oxysporum* in greenhouse experiments. We analyzed tomato gene expression connected to salicylic acid (SA), jasmonic acid (JA), and ethylene (ET) signaling pathways after *B. cinerea* infection to determine potentially activated defense responses.

**Results:**

The results showed that *K. radicincitans* and *P. phytofirmans* significantly inhibited the mycelial growth of *F. oxysporum* in dual culture assays, while only *K. radicincitans* inhibited *B. cinerea* mycelial growth via diffusible compounds; volatile metabolites had negligible antifungal effects for both strains. In the *Botrytis* greenhouse experiment, *P. phytofirmans* significantly reduced gray mold disease symptoms in detached-leaf assays and enhanced root biomass, whereas *K. radicincitans* conferred neither effect. Gene expression analyses showed no consistent induction of JA- or ET-pathway marker genes across treatments. Notably, *SlPR1a*, an SA-pathway marker, was more strongly induced in *P. phytofirmans*-colonized plants upon *B. cinerea* challenge compared to *K. radicincitans*-inoculated and non-inoculated controls, a pattern consistent with, though not sufficient to confirm, a priming-associated mechanism. Formal demonstration of priming would require time-course analyses of defense gene kinetics, which were not conducted in this study. In the Fusarium greenhouse experiment, neither strain significantly suppressed vascular wilt disease or restricted *F. oxysporum* colonization in tomato stems, despite demonstrating *in vitro* antagonism, underscoring the well-documented disconnect between *in vitro* and *in planta* biocontrol performance.

**Discussion:**

These findings indicate that *in vitro* antagonism poorly predicts *in planta* biocontrol performance. *Paraburkholderia phytofirmans* shows promise for indirectly controlling tomato gray mold, likely associated induction of systemic defense responses.

## 1 Introduction

Tomato (*Solanum lycopersicum* L.) is one of the most cultivated crops globally (FAOSTAT, 2024) and serves as a model crop in plant science (Kimura and Sinha, 2008) and in investigating the interactions between plants and microbes (Liu et al., 2022; Orine et al., 2025). Ripe fruits are nutritionally rich and economically significant worldwide (Nishino et al., 2002; Saltveit, 2005). Among the many determinants of tomato health and growth, beneficial bacterial endophytes have garnered significant attention for their beneficial roles under abiotic and biotic stress (Zampieri et al., 2023; Abbas et al., 2024; Fagnano et al., 2025). Therefore, beneficial plant-associated microbes have emerged as key contributors to plant growth, development, and stress resilience.

Plant growth-promoting bacteria (PGPB) enhance growth through nutrient conversion or indirectly phytohormone production and protection from biotic stresses such as phytopathogens, either directly through antagonism or indirectly by activating inducible systemic resistance (ISR) and modulating the balance of phytohormones (Choudhary and Varma, 2016; Tsukanova et al., 2017; Timofeeva et al., 2023; Etesami, 2025). Similar beneficial effects have been documented in tomato (Mayak et al., 2001; Berger et al., 2017; Yakti et al., 2018). Against this background, *Kosakonia radicincitans* has appeared as a well-studied model organism exhibiting pronounced growth-promoting and protective functions in various crop plants, including tomato.

*Kosakonia radicincitans* (family: Enterobacteriaceae) is a gram-negative, and motile endophytic bacterium (Kämpfer et al., 2005; Brady et al., 2013; Becker et al., 2018), isolated from the phyllosphere of winter wheat (Ruppel, 1987). It promotes growth and yield of tomato (Berger et al., 2013; 2017), fixes nitrogen, solubilizes phosphorus (Schilling et al., 1998), induces plant resistance (Brock et al., 2013), and inhibits the growth of fungal phytopathogens (Lambrese et al., 2018). The strain DSM 16656 confers the plant resistance against phloem-feeding and chewing insects (Brock et al., 2018) and protects fungal hyphae against a bacterial predator (Sharma et al., 2021).

*Paraburkholderia phytofirmans* (family: Burkholderiaceae) is a gram-negative, motile, and epi- and endophytic bacterium (Sessitsch et al., 2005; Mitter et al., 2013; Sawana et al., 2014) isolated from surface-sterilized roots of onion plants (Frommel et al., 1991a). It promotes tomato growth (Pillay and Nowak, 1997; Issa et al., 2018; Galambos et al., 2020), induces auxin phytohormone production (Naveed et al., 2015), primes early ISR (Timmermann et al., 2019), lessens disease symptoms caused by *Pseudomonas syringae* pv. tomato DC3000 in *Arabidopsis thaliana* (Timmermann et al., 2017) and *Xylella fastidious* on grapevine (Baccari et al., 2019). Relevant to the present study, this strain primes early ISR against *B. cinerea* in *A. thaliana* (Timmermann et al., 2019; Nešić et al., 2025) and suppresses the growth of *B. cinerea* in vineyards (Miotto-Vilanova et al., 2016).

*Botrytis cinerea* is a necrotrophic, airborne phytopathogenic fungus causing gray mold disease in a wide range of hosts including greenhouse vegetables (Toral et al., 2020; Singh et al., 2024). In tomato, gray mold accounts for pre- and post-harvest losses of 20-50% of annual fruit production, with some estimates exceeding 80% under severe conditions (Syed Ab Rahman et al., 2018; Rhouma et al., 2023). Across all crops, annual economic losses attributable to *B. cinerea* are estimated at USD 10–100 billion worldwide (Roca-Couso et al., 2021). The infection phases of *B. cinerea* typically include host surface penetration, tissue death (initial lesion development), tissue maceration (lesion expansion), and spore generation (van Kan, 2006; Choquer et al., 2007). Because of its wide host range, multiple invasion modes, and both asexual and sexual stages of survival, controlling *B. cinerea* spread remains challenging (Fillinger and Elad, 2016; Hua et al., 2018). As an airborne necrotroph that rapidly kills host tissue, *B. cinerea* is frequently used as a model pathogen to evaluate induced systemic resistance (ISR) mechanisms targeting cell-death-associated infections (Singh et al., 2024).

*Fusarium oxysporum* f.sp. *lycopersici* is a hemibiotrophic, soil-borne fungal pathogen, causing vascular wilt disease in tomato characterized by yellowing leaves, browning vascular stems, wilting plant stems, and ultimately the plant death (Lim et al., 2006; Vinchira-Villarraga et al., 2021). Yield losses range from 30-45% under typical conditions to 70-80% in severe cases (Nirmaladevi et al., 2016; Srinivas et al., 2019), contributing substantially to global losses, exceeding US $1 billion annually (Ghazalibiglar et al., 2016; Ansari et al., 2024). *Fusarium oxysporum* is classified into three races based on compatibility with tomato resistance genes. Race 3 is particularly problematic because it overcomes common resistance genes (*I* and *I-2*) (Swett et al., 2023), making systemic disease management especially difficult.

The two pathogens studied here, *B. cinerea* and *F. oxysporum* f. sp. *lycopersici* race 3, were deliberately selected because they are phylogenetically and ecologically distinct, employing fundamentally divergent infection strategies: *B. cinerea* is an airborne necrotroph that causes rapid cell death and establishes infection primarily through foliar and fruit surfaces, whereas *F. oxysporum* f. sp. *lycopersici* is a soilborne hemibiotroph that colonizes root tissue and invades the vascular system systemically (Takken and Rep, 2010; Dean et al., 2012). This contrasting pathosystem design allows us to evaluate a central unresolved question in biological control research: whether bacterial-mediated resistance in tomato operates in a pathogen-specific manner or confers broad-spectrum protection regardless of pathogen lifestyle, infection route, or ecological niche. Addressing this question has critical implications for the development of durable, multi-target biocontrol strategies (Pieterse et al., 2014; Ansari et al., 2024).

Plant immunity involves recognition of pathogen-associated molecular patterns triggering PTI, followed by effector-triggered immunity (ETI) mediated by resistance (R) proteins (Jones and Dangl, 2006). Defense signaling is orchestrated primarily through salicylic acid (SA), jasmonic acid (JA), and ethylene (ET) pathways, which activate pathogenesis-related (PR) proteins and other antimicrobial responses (Robert-Seilaniantz et al., 2011; Pieterse et al., 2014). SA-mediated systemic acquired resistance (SAR) is generally associated with defense against biotrophic pathogens, while JA/ET-mediated ISR is typically induced by beneficial root bacteria and confers resistance against necrotrophs such as *B. cinerea* (Grant and Jones, 2009). ISR does not always involve overexpression of PR proteins but can instead operate via priming, a state of heightened readiness in which plants mount faster or stronger defense responses upon pathogen attack (Conrath et al., 2006; Tsalgatidou et al., 2023).

A critical and frequently unresolved challenge in biological control research is that *in vitro* antagonism assays do not reliably predict biocontrol efficacy under plant-associated conditions (Fravel, 2005; Besset-Manzoni et al., 2019; Lahlali et al., 2022). Discrepancies between laboratory and greenhouse performance are commonly attributed to differences in rhizosphere competence, environmental regulation of secondary metabolite production, and competition with native soil microbiota (Weller, 1988; Pliego et al., 2011; Gómez Expósito et al., 2017). The present study does not aim to resolve the mechanistic basis of this discrepancy but rather uses *in vitro* and *in planta* assays in parallel as an observational comparison to assess the overall biocontrol potential of two PGPB candidates.

However, the ability of *K. radicincitans* and *P. phytofirmans* to induce plant resistance against pathogenic *B. cinerea* or *F. oxysporum* in tomato plants has not previously been tested in direct comparison. This work aims to evaluate the potential of *K. radicincitans* or *P. phytofirmans* to be used as biocontrol agents (BCAs) by assessing (i) *in vitro* antagonistic activity, (ii) inducing systemic resistance, and (iii) plant growth promotion under stressed and non-stressed conditions.

## 2 Materials and methods

### 2.1 Cultures of microorganisms

#### 2.1.1 Kosakonia inoculum

ABiTEP GmbH, Berlin, kindly provided *Kosakonia radicincitans* DSM 16656 in a lyophilized formulation, which we stored at -80 °C. To find out how many bacteria were in one gram of the lyophilized sample, we mixed it with 10 mL of sterile 10 mM MgCl_2_ buffer, spread it on a special bacteria growth medium (523 from Duchefa Biochemie B.V., Haarlem, The Netherlands), and kept it at 30 °C for two days. The original stock was equivalent to 10^10^ colony-forming units per gram (CFU gram^-1^). Aliquots were then prepared and stored at -20 °C for later use. The final inoculum concentration was diluted to 10^8^ CFU mL^-1^ by sterilized MgCl_2_ buffer, and it was confirmed with a single plate count (Thomas et al., 2015) on bacteria screening medium 523 at 24 °C for 24 hours.

#### 2.1.2 Paraburkholderia inoculum

*Paraburkholderia phytofirmans* PsJN was kindly provided by Dr. Angela Sessitsch (Austrian Institute of Technology GmbH, Vienna) on Petri dishes with a standard nutrient medium. The bacterial strains were stored long-term in -80 °C at 80% glycerol solution. For the inoculum preparation of the bacteria, two loops of *P. phytofirmans* were inoculated into 100 mL of LB broth in a 250 mL Erlenmeyer flask and incubated at 30 °C for 24 hours at 180 rpm (Mitter et al., 2017). The bacterial suspension was centrifuged at 3,000 rpm for 10 minutes at 4 °C (Compant et al., 2005), and the cells were pelleted and washed twice with 10 mM MgCl_2_ buffer (pH = 6) (Su et al., 2017). The bacterial pellet was resuspended by vortexing. One mL of the final bacterial suspension contained 9 × 10^8^ CFU, which was equivalent to an optical density of 1.7 at 600 nm (DS-11 Series Spectrophotometer/Fluorometer; DeNOVIX, Wilmington, DE, USA). This suspension was adjusted to 10^8^ CFU mL^-1^ and confirmed with a single plate count (Thomas et al., 2015) by plating on King’s B medium and incubating at 30 °C for 48 hours.

#### 2.1.3 Botrytis inoculum

**Figure 1:**
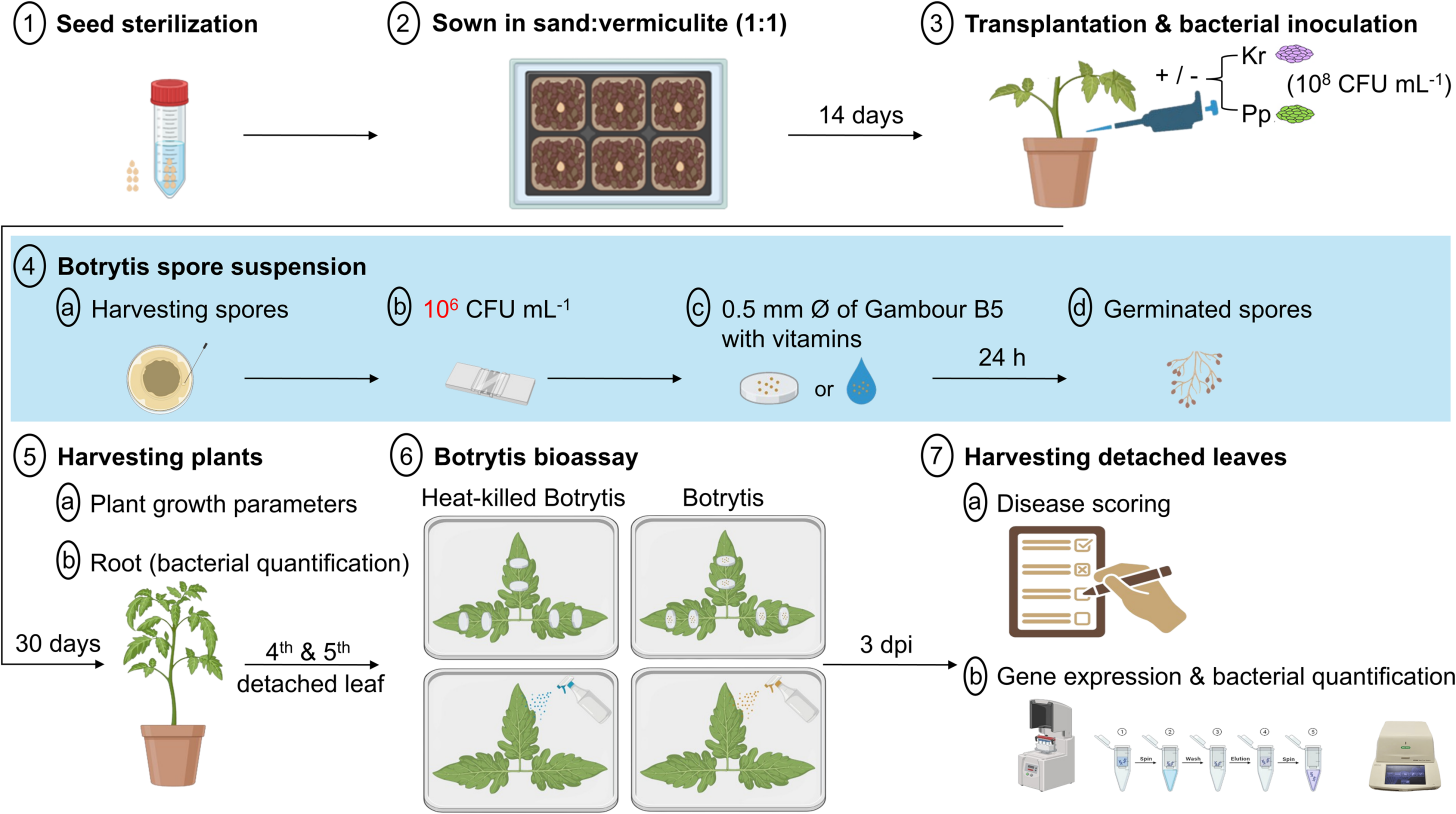
Schematic diagram of the Botrytis experiment. On day 1, tomato seeds were surface sterilized with ethanol (99%) for 1 minute, sodium hypochlorite (10%) for 5 minutes, and rinsed three times in sterile distilled water. Seeds were placed on sterilized mixed sand and vermiculite (1:1), growing until the first and second true leaves appeared (2 weeks). After 14 days, seedlings were then transplanted into 1 L pots and inoculated with one mL of 1 × 10^8^ CFU of endophytic bacteria *Kosakonia radicincitans* (Kr) or *Paraburkholderia phytofirmans* (Pp) or 10 mM MgCl_2_ buffer as mock controls. Seedlings were grown for 30 days as a colonization period. One day before the challenge with *Botrytis cinerea* on day 43, spore suspension was harvested from 3-week-old *B. cinerea* grown on PDA plates. Then the suspension was filtered with autoclaved cheesecloth, adjusted to 10^6^ CFU mL^-1^ in Gambour B5 liquid with supplemented 10 mM sucrose and 10 mM KH_2_PO_4_, and stored overnight at room temperature for spore germination. Furthermore, the plugs of Gambour B5 with vitamins of 5 mm diameter have been pipetted with 10 µL of 10^6^ CFU mL^-1^ or autoclaved spore suspension as mock inoculation (heat-killed *B. cinerea* spores) and stored overnight at room temperature for the bioassay experiment. Plant growth parameters like height and number of leaves were scored. On day 44, plants were harvested, and samples from roots were stored at -80 °C for extraction of genomic DNA to verify the colonization of the bacteria. Three terminal leaflets of the fourth and fifth leaves of each plant were detached for challenging the plugs and spore suspension, respectively. The rest of the shoots were harvested for plant growth parameters. After three days post-incubation (dpi) on a 4-layer tissue paper in a 145-mm-diameter Petri dish and moistened with sterile distilled water on day 47, disease scoring was determined by calculating the average of measurements of the tallest and shortest diameters of the lesion. Leaf samples were stored at -80 °C for the extraction of gDNA and RNA to determine bacterial colonization and target genes, respectively. The figure was created with BioRender software (https://www.biorender.com).

*Botrytis cinerea* (strain: DSM 4709) was provided by the German Collection of Microorganisms and Cell Cultures (DSMZ). Potato extract glucose agar (PDA) plates (Carl Roth, Karlsruhe, Germany) were used to develop, maintain, and subculture *B. cinerea* every three weeks at pH 5.6 ± 0.2 (Zhang et al., 2013). The fungus was cultivated for two weeks at 24 °C in the dark and one week in daylight at room temperature (23 °C). To harvest spores, *B. cinerea* was grown on half-strength potato dextrose medium at 25 °C for 10–12 days (Ren et al., 2008) with 1.5% agar (Zhang et al., 2013). Spores were collected by a plastic spatula after flooding the plates with 7 mL of sterile tap water. *Botrytis cinerea* spores were cultured in Gambor’s B5 medium (Duchefa, Haarlem, The Netherlands) blended with 0.1 mM sucrose and 0.1 mM KH_2_PO_4_ for 4 h in the dark without shaking (Vicedo et al., 2009; Martínez-Medina et al., 2013). The spore suspension was then filtered through sterile cheesecloth, measured using a hemacytometer, and adjusted to 10^6^ spores mL^–1^ (Day 43, **Figure 1**).

#### 2.1.4 Fusarium inoculum

*Fusarium oxysporum* f. sp. *lycopersici* (FOL 029, race 3) was kindly provided by Dr. Frank Takken (Swammerdam Institute for Life Sciences, Amsterdam) on PDA plates. It was maintained and subcultured at 25 °C for 10 days in the dark (Abbasi et al., 2019). The surface of the fungal cultures was then flooded with 5 or 10 mL of sterile tap water per plate and surface-scraped using a plastic spatula to collect spores. Conidial spores were collected by filtering the culture through four layers of autoclaved cheesecloth (Prihatna et al., 2018). Spore concentration was measured using a hemacytometer, and it was then diluted to 10^7^ spores mL^-1^ (van der Does et al., 2019).

### 2.2 *In vitro* antagonist experiment

The antagonistic effect of beneficial microbes against fungal pathogens is usually due to the release of diffusible secondary metabolites and volatile compounds (Syed-Ab-Rahman et al., 2019). To illustrate the reason behind the antagonism, the effect of diffusible secondary metabolites was evaluated by using dual culture plates, while the influence of volatile organic compounds was investigated by using two-compartment plastic plates (Fürnkranz et al., 2012). We tested the *in vitro* antifungal activities of the two PGPB, *K. radicincitans* and *P. phytofirmans*, against two fungal phytopathogens: *B. cinerea* and *F. oxysporum*. All antifungal assays were performed in Petri dishes (90 * 15 mm; Sarstedt AG, Nürnbrecht, Germany), which contained one tested PDA medium for all fungi and bacteria (**Supplementary Figure 1**). Each experiment included three replicates of each bacterial strain and control. Images of the mycelium were taken with an Epson Perfection V850 Pro scanner using the Epson Scan Windows application Version 3.9.3.4 (US) with 24-bit color and 300 dpi resolution (Epson Europe, Amsterdam, The Netherlands).

#### 2.2.1 Dual culture

To examine the antagonism based on diffusible compound effects, a bacterial suspension of *K. radicincitans* or *P. phytofirmans*, 20 µL of the 10^8^ CFU mL^-1^ in sterile 10 mM MgCl_2_ buffer solution, was inoculated at 24 °C at the two opposite sides, 1 cm from the plate edge, of PDA plates (**Supplementary Figure 1A**). As mock controls, 20 µL of sterile 10 mM MgCl_2_ buffer solution was pipetted. Petri dishes were sealed with Parafilm membrane (Bemis flexible packaging, Neenah, WI) and incubated in darkness at 24 °C. Two days later, an 8 mm diameter fungal plug from 28-day-old plates of *B. cinerea* or *F. oxysporum* was then placed in the center of the same medium plate, followed by incubation at 24 °C in the dark for 10 days for *B. cinerea* (Alenezi et al., 2017) and for 4 days for *F. oxysporum* (Abbasi et al., 2019). The antagonism was determined by estimating the inhibition of mycelial fungal growth percentages. The percentage was calculated using the formula [(a − b)/a] × 100, where “a” is the growth radius of a control culture (in cm) and “b” is the distance of the pathogen growth in the direction of the bacteria (in cm) (Trivedi et al., 2008).

#### 2.2.2 Two-compartment dish

To test the antagonism based on volatile compound mechanisms, two-split Petri dishes were loaded with the same PDA medium for the two compartments (**Supplementary Figure 1B**). One half was pipetted with 20 µL of the prepared bacterial suspension in the middle of the compartment and incubated in the dark at 24 °C. After 48 h, a fungal plug of an 8 mm diameter was placed in the middle of the other half and incubated at 24 °C in darkness for 10 and 4 days for *B. cinerea* and *F. oxysporum*, respectively. The estimation of the antagonism and photographing the plates were conducted as aforementioned.

### 2.3 Botrytis greenhouse experiment

#### 2.3.1 Plant materials

**Figure 2:**
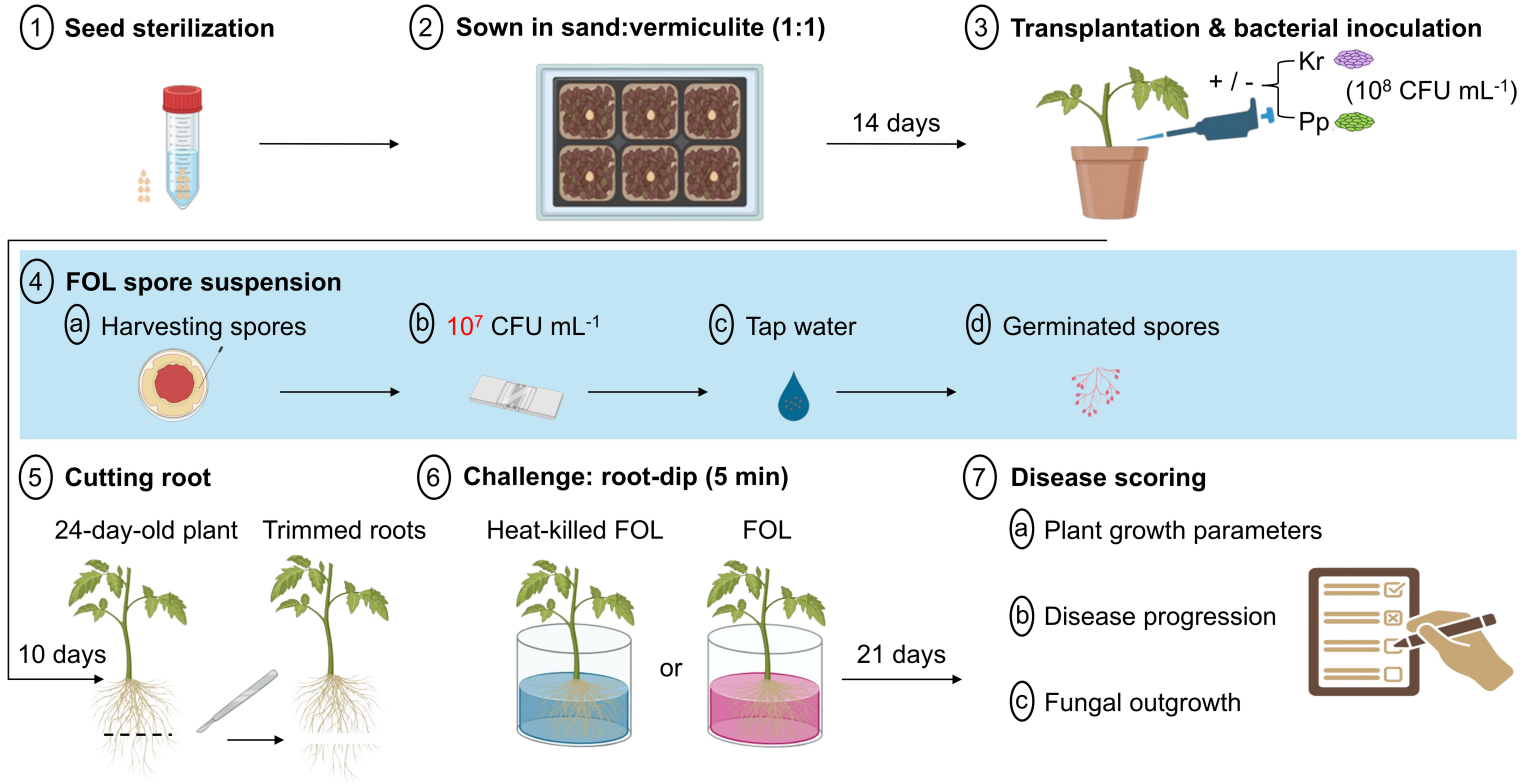
Schematic diagram of the Fusarium experiment. on day 1, tomato seeds were surface sterilized with ethanol (99%) for 1 min, sodium hypochlorite (10%) for 5 mins, and rinsed three times in sterile distilled water. Seeds were placed on sterilized mixed sand and vermiculite (1:1), growing until the first and second true leaves appeared (2 weeks). After 14 days, seedlings were then inoculated with 1 mL of 1 × 10^8^ CFU of endophytic bacteria *Kosakonia radicincitans* (Kr), *Paraburkholderia phytofirmans* (Pp), or 10 mM MgCl_2_ buffer as mock controls. Seedlings were grown for 10 days as a colonization period. One day before the challenge with *Fusarium oxysporum* f.sp. *lycopersici* (FOL) on day 23, conidial spores of *F. oxysporum* were harvested by scraping in sterile water (10 mL per plate) and final conidia mL^-1^ and filtered with sterilized cheesecloth, adjusted to 10^7^ using a hemocytometer. Then, tomato seedlings were uprooted, trimmed, and challenged with a conidial suspension of *F. oxysporum* for 5 min (root-dip method) while seedlings were submerged in an autoclaved spore suspension as mock inoculation (heat-killed FOL spores) as a negative control. Thereafter, seedlings were then transplanted into 1-L pots to grow for 3 weeks as a part of the challenge period. After 21 days, on day 45, plant growth parameters like height, number of leaves per plant, and biomass were measured. Plants were then harvested, and samples from the cotyledon and stem were used to estimate disease progression. Tomato plants were cut at soil level, and leaves were removed. Entire plants were subsequently surface sterilized by submergence in 70% ethanol and washed by submergence in sterile water. A slice of each sterilized stem piece was put on a plate made of PDA, supplemented with 200 mg L^-1^ streptomycin and 112 mg L^-1^ penicillin to reduce bacterial growth for 2 days post-incubation (dpi) in the dark at 25 °C. After 2 dpi on day 47, fungal outgrowth was scored. The figure was created with BioRender software (https://www.biorender.com).

Tomato seeds (*Solanum lycopersicum* L. cv., “Moneymaker”) were bought from Kings Seeds (Essex, UK) and surface sterilized with ethanol (99%) for 1 minute and sodium hypochlorite (10%) for 5 minutes, rinsed three times in sterile distilled water, and left to dry. Seeds were then planted in a sterile commercial substrate (Einheitserde type VM: Vermehrungserde, Germany) for germination in a 72-cell seed starting tray (Day 1, **Figure 1**; **Figure 2**). Seedlings were watered every day with tap water and kept under greenhouse conditions. The controlled conditions of the greenhouse were 20–25 °C under a 16 h light and 8 h dark cycle and 65% relative humidity (RH). Uniform seedlings were individually transplanted into one-liter pots (15 × 20 cm) filled with a mixture of sterilized vermiculite (Isola Vermiculite, Sprockhövel, Germany) and coarse sand (Euroquarz GmbH; Ottendorf-Okrilla, Germany) (1:1) after two weeks (Day 14, **Figure 1**; Day 24, **Figure 2**). When needed, plants were irrigated with tap water, while they were fertilized three times a week with 100 mL of a 1:100 solution of a soluble fertilizer possessing an EC value (Kristalon™, NPK 18-18-18, Yara Ltd., Vlaardingen BV, Netherlands) (Jafari et al., 2016).

#### 2.3.2 Inoculation with bacteria and Botrytis challenge

After transplantation (Day 14, **Figure 1**), 14-day-old tomato seedlings were immediately inoculated with one mL of 10^8^ CFU mL^-1^ of either *K. radicincitans* or *P. phytofirmans*. Control seedlings were treated with one mL of 10 mM MgCl_2_ buffer as mock inoculation. Using a micropipette, the bacterial suspensions or mocks were delicately dispersed in the soil close to the roots. Each treatment had 10 biological replicates. After 30 days as a colonization period, growth parameters, including fresh and dry shoots and root weight, shoot length, and quantity of leaves, were measured and recorded.

#### 2.3.3 Botrytis bioassays

The main objective of this experiment was to estimate the long-term effect of each bacterial strain against *B. cinerea* on tomato plants. Detached-leaf assays were used to assess whether distal leaves had acquired an altered defensive state following root inoculation with PGPB, rather than to monitor active systemic signaling after pathogen challenge. This approach was selected because it allows standardized quantification of resistance phenotypes in excised tissue under controlled inoculation conditions (Martínez-Medina et al., 2013; Martínez-Hidalgo et al., 2015). Therefore, the adaxial surface of detached tomato leaves was challenged by either plugs or spore suspension (10^6^ conidial spores mL^–1^) after one month of bacterial inoculation (Day 44, **Figure 1**). To minimize variability inherent to the detached-leaf assay, leaves of identical developmental stage and position (terminal three leaflets of the fourth true leaf) were selected across all replicates, and experiments were conducted across five independent biological replicates. To determine the disease damage of the *B. cinerea* challenge, 10 µL of 10^6^ CFU mL^-1^ or sterile water has been pipetted onto a 5-mm-diameter plug of Gambour B5 with vitamins medium (Duchefa, Haarlem, The Netherlands). This concentration of 10^6^ conidia mL^−1^ for *B. cinerea* was selected based on widely established detached-leaf assays in tomato, where this inoculum density reliably induces reproducible necrotic lesions without causing overwhelming tissue maceration (Martínez-Hidalgo et al., 2015; You et al., 2023). Plugs were stored overnight at ambient room temperature one day ahead of challenging leaves. Conidial spores were checked for germination under the microscope.

The terminal three leaflets of the fourth true leaves were detached by a scalpel, and they were then infested with germinated conidial *B. cinerea* plugs. Plugs without mycelium were used for the mock test. Two 5-mm-diameter plugs per leaflet were then incubated in a 145-mm-diameter square plastic dish (Greiner Bio-One, Frickenhausen, Germany) with four layers of wetted sterile-paper tissue. Plastic dishes were incubated in a climate chamber at 21 °C with constant light and 70% RH for 72 hours as an incubation period. Disease damage was scored (Day 47, **Figure 1**) by measuring the diameter of lesions by computing the average of the longest and shortest diameters of each necrotic lesion 72 h post-pathogen inoculation with ImageJ/Fiji software (NIH, v2.10). We recognize that detached leaf assays do not preserve whole-plant physiological integration, including resource allocation and continuous vascular signaling. Consequently, the results of this bioassay should not be interpreted as direct evidence of active systemic signaling during infection, but rather as evidence that root inoculation is associated with enhanced resistance in distal leaf tissue under detached-leaf conditions. Furthermore, after 72 h of challenging with *B. cinerea* plugs, disease damage levels were marked by measuring the extension of gray mold lesions outside plugs in the leaflets. These levels were classified into three divisions: 1–2 mm from plugs as mild; 3–5 mm from plugs as moderate; more than 5 mm as severe (Martínez-Hidalgo et al., 2015). Each treatment had five biological replicates. The biocontrol effectiveness of each bacterial strain was determined as [(lesion diameter in control - lesion diameter in treatment)/lesion diameter in control] × 100 (Wang et al., 2020).

#### 2.3.4 Quantification bacteria of colonized plant tissues

##### 2.3.4.1 Plant DNA extraction

To detect *K. radicincitans* or *P. phytofirmans* in plant roots and leaves, the gDNA of plant materials was extracted using the DNeasy Plant Mini Kit (Qiagen, Hilden, Germany), following the manufacturer’s instructions. Leaf samples, approximately 100 mg of the terminal leaflet of the fourth true leaf, were initially ground to a fine powder with 1.4- and 2.8-mm ceramic beads (1:1) inside two-mL Precellys tubes, which were cooled via liquid nitrogen via the Cryolys device attached to the Precellys^®^24 homogenizer (Bertin Instruments, Montigny-le-Bretonneux, France). Samples were homogenized at 6500 rpm for 2x40 sec and for 10 sec at -10 °C. DNA was eventually eluted in 100 µL DEPC-treated water and stored at -20 °C before use.

The DNA yield (ng) and purity (absorbance ratio at 260/280) of extracted gDNA were determined spectrophotometrically using 1 µL of gDNA in the DS-11 Series Spectrophotometer/Fluorometer (DeNOVIX, Wilmington, DE, USA), where pure DNA is defined as having a 260/280 absorbance ratio ranging between 1.7 and 2.0 (Chen et al., 2010). The integrity of gDNA was determined by visualizing approximately 200 ng of DNA (5 µL of eluted DNA mixed with 1 µL of loading bromphenol blue and sucrose (BBS) gel dye per well on a 1% agarose gel (w/v) containing 0.25 μg/μL of ethidium bromide (EtBr), run in 1× TAE buffer (Jena Bioscience, Germany) at 100 V for 40 minutes with gel electrophoresis (VWR^®^ Mini Gel, Leuven, Belgium). The extracted DNA sizes were estimated using the DNA marker 1 kb Plus DNA Ladder (Thermo Scientific, Graiciuno, Vilnius, Lithuania). A digital image was taken under UV light in the FAS-V Gel Documentation System (CCD-Sensor) (Nippon Genetics, Dueren, Germany).

##### 2.3.4.2 Bacterial DNA extraction

Lyophilized *K. radicincitans* or pellets of *P. phytofirmans*, approximately 10^8^ CFU, were pretreated by adding 3.6 mg of lysozyme to 180 µL of enzymatic lysis buffer, which consisted of 20 mM tris-HCl (pH = 8.0), 2 mM sodium EDTA, and 1.2% Triton, and incubated at 37 °C for 30 min with a rotator. DNA extraction was conducted according to the manufacturer’s instructions for DNeasy Blood and Tissue Kits (Qiagen, Hilden, Germany) designed for Gram-positive bacteria. 100 µL of PCR water was used to elute the extracted gDNA. DNA dilution series were then created and stored at -20 °C. These DNA series were employed for absolute quantification.

##### 2.3.4.3 Detection bacteria in plants

The presence of *K. radicincitans* or *P. phytofirmans* in plant roots and leaves was checked by amplifying relative genomic DNA (gDNA) in the samples by quantitative PCR (qPCR). The *K. radicincitans* were detected and quantified by using a specific primer pair (fdnaJ-F1 and fdnaJ-R1), amplifying a product of 140 bp (Witzel et al., 2017) (**Supplementary Table 1**). qPCR reactions contained (10 µL total volume): 1x SsoAdvanced Universal SYBR Green Supermix (BioRad, Feldkirchen, Germany), 0.2 µM of each primer, and 5–100 ng of DNA. The qPCR was run at the following settings: hot start at 95 °C for 3 min, 35 cycles of 95 °C for 15 sec, followed by 50 sec at 72 °C, and 5 min of elongation time at 72 °C. *Paraburkholderia phytofirmans* were detected and quantified by using the probe and primers designed in the previous study (Sheibani-Tezerji et al., 2015) to match the gene for transcription termination factor rho (Bphyt_1824) in the genome of strain PsJN (**Supplementary Table 1**). qPCR reactions comprised 10 µL total volume: 1x SsoFast Probes (BioRad, Feldkirchen, Germany), 0.5 µM of each primer, 0.35 µM probe, and 5–100 ng of DNA. The qPCR was run at the following settings: hot start at 95 °C for 2 min, 40 cycles: denaturation at 95 °C for 5 sec, and hybridization and elongation for 20 sec at 59 °C (Mitter et al., 2017). All qPCR data analysis was performed using CFX Maestro™ software (BioRad, Feldkirchen, Germany). To visualize qPCR products, amplicons were mixed with loading dye in a 1% agarose gel (w/v) containing 0.25 µg/µL of EtBr in 1x TAE buffer at 80 volts for 40 minutes.

#### 2.3.5 RNA extraction and qRT-PCR

We tested whether the colonization of PGPB strains affects the expression of defense-related genes in the absence or presence of *B. cinerea* in detached leaves. *Botrytis cinerea* spore suspension (10^6^ mL^-1^), or autoclaved spores, was sprayed on the terminal three leaflets of the fifth true leaf, which had been inoculated with bacterial treatments for one month before pathogen challenge. Leaves were then kept at high humidity for 72 hours. The 72-hour post-challenge time point was selected because it corresponds to the biologically relevant stage at which necrotic lesion development by *B. cinerea* is visually established and reproducibly quantifiable in detached-leaf assays (Martínez-Hidalgo et al., 2015; You et al., 2023), and at which defense gene induction in response to this necrotroph has been previously documented in tomato (AbuQamar et al., 2008). We recognize that this single time point precludes assessment of the kinetics of defense gene induction, which is a prerequisite for formally demonstrating priming (Conrath et al., 2006). Time-course analyses at multiple post-infection intervals are therefore identified as a priority for future work (see Section 5.4). These conditions helped the pathogen be exposed to the leaf tissue uniformly and concurrently in order to obtain evenly distributed and accurate gene expression patterns (Martínez-Hidalgo et al., 2015). Moreover, we estimate “priming”, the physiological state in which pre-inoculation with beneficial PGPB prepares the plant to mount a faster or stronger defense response upon subsequent infection with *B. cinerea* spores. The RNA extraction procedure from tomato leaves was performed using the RNeasy Mini kit (Qiagen, Düsseldorf, Germany) in accordance with the manufacturer’s instructions. To ensure RNA integrity, DEPC-treated water (Ambion, cat. no. AM9906, Thermo Fisher Scientific, Dreieich, Germany) was used in the purification steps. Total RNA was further purified by DNase treatment (Qiagen, Hilden, Germany). Complementary deoxyribonucleic acid (cDNA) synthesis was subsequently processed using 1 µg of each purified total RNA, employing the QuantiTect^®^ Reverse Transcription Kit (DNase I & transcript) (Qiagen, Hilden, Germany). The resulting cDNA was stored at -80 °C until further analysis.

Before using generated cDNA for gene expression assays, the success of the purification step of total RNA and the selection of reference genes was confirmed by PCR, comparing their amplicons with relative total RNA, purified RNA, and tomato gDNA as controls. Successful purified RNA should not exhibit any amplicon, but cDNA and gDNA should clearly exhibit different amplicon sizes (**Supplementary Figure 2**). PCR products were observed on agarose gel electrophoresis by using specific primer pairs of six reference genes obtained with the specific primer pairs of tomato housekeeping genes: ubiquitin 3 (*SlUbi3*), elongation factor 1α (*SlEF*), actin (*SlACT*), glyceraldehyde 3-phosphate dehydrogenase (*SlGAPDH*), tubulin (*SlTUB*), and α-tubulin (*SlTUB α*) (**Supplementary Table 2**). The PCR reaction contained 0.2 µM of each primer, 1x Mytaq^®^ buffer (Bioline, Luckenwalde, Germany), and 1 unit of MyTaq™ HS DNA polymerase (Bioline), with 1 µL of template in a final volume of 10 µL. The thermal cycler was set to run for an initial 2 minutes at 95 °C, followed by 40 cycles of 15 seconds at 95 °C, and then one minute of annealing and extension at 60 °C. Amplicons were tested by electrophoresis. Wells loaded with 10 µL of PCR product after mixing with loading dye. Electrophoresis was conducted on a 1% agarose gel (w/v) containing 0.25 µg/µL of EtBr in 1x TAE buffer at 80 volts for 1 hour.

Quantitative reverse-transcription polymerase chain reaction (qRT-PCR) experiments were conducted to detect upregulation or downregulation of plant defense genes for six treatments: uninoculated, inoculated plants with *K. radicincitans*, and inoculated plants with *P. phytofirmans* in the absence of pathogen (mock) or the presence of *B. cinerea*. Six defense genes from tomato plants were selected (**Supplementary Table 3**), encoding pathogenesis-related protein 1a (*SlPR1a*) (Gen ID: Solyc01g106620.2) (Martínez-Medina et al., 2013), WRKY transcription factor 70 (*SlWRKY70*) (Gen ID: Solyc05g014040) (Abbasi et al., 2019), proteinase inhibitor II (*SlPin II*) (Gen ID: Solyc03g020060) (Uppalapati et al., 2005), lipoxygenase A (*SlLoxA*) (Gen ID: Solyc08g014000) (López-Ráez et al., 2010), defensin-like proteins 4 (*SlDEF4*) (Gen ID: Solyc07g009060.4.1) (Nikoloudakis et al., 2020), and ethylene response factor 1 (*SlERF1*) (Gen ID: Solyc08g078180) (Abbasi et al., 2019). These six genes were selected because they represent well-validated transcriptional markers for the three principal hormone-mediated defense signaling pathways in tomato: the salicylic acid (SA) pathway (*SlPR1a*, *SlWRKY70*), the jasmonic acid (JA) pathway (*SlPin II*, *SlLoxA*), and the ethylene (ET)-associated pathway (*SlDEF4*, *SlERF1*). This three-pathway coverage was chosen because SA-, JA-, and ET-mediated signaling are the primary routes through which ISR and SAR operate in response to necrotrophic and hemi-biotrophic pathogens (Robert-Seilaniantz et al., 2011; Pieterse et al., 2014), and because the selected markers have been previously applied in analogous tomato–PGPB interaction studies (Martínez-Medina et al., 2013; Abbasi et al., 2019). We acknowledge that a broader marker set, including genes encoding secondary metabolite biosynthesis enzymes or additional *WRKY* transcription factors, could provide a more granular view of the transcriptional reprogramming. However, expansion of the marker set was beyond the scope of the present study, which was designed as an initial comparative screen of two PGPB candidates across two contrasting pathosystems. The housekeeping genes *SlACT* (Gen ID: Solyc11g005330) and *SlGAPDH* (Gen ID: Solyc04g009030) were used as reference genes after showing high expression stability (**Supplementary Table 2**). Expression values were normalized using the geometric mean of the two reference genes. Three independent biological replicates were analyzed per treatment, and each qPCR reaction was performed in triplicate as technical replicates. The qPCR reaction contained 0.2 µM of each primer, 2x qPCR S’Green BlueMix (Biozym, Hessisch Oldendorf, Germany), and 1 µg of cDNA template in a final volume of 20 µL. Thermal cycler: initially 2 min at 95 °C, then 40 cycles at 95 °C for 5 sec, followed by annealing and extension for 30 sec at 60 °C.

### 2.4 Fusarium greenhouse experiment

#### 2.4.1 Plant materials and greenhouse conditions

In this experiment, *K. radicincitans* and *P. phytofirmans* were tested for their ability to confer resistance against pathogenic *F. oxysporum*. The tomato plant (*S. lycopersicum*), specifically the “Moneymaker” variety, is vulnerable to race 3 of *F. oxysporum* (Catanzariti et al., 2015), and was utilized for conducting disease assays using the two PGPB. After sowing sterilized seeds in sterile mixed soil as mentioned above, seedlings were cultivated in a greenhouse with a day-night temperature range of 20–25 °C, 16 hours of light followed by 8 hours of darkness, and a RH of 65%.

#### 2.4.2 Inoculation with bacteria and Fusarium challenge

14-day-old tomato seedlings were inoculated with bacteria (one mL with 10^8^ bacteria for each strain on the soil surface) (Day 14, **Figure 2**). Ten days later, the plants were challenged by *F. oxysporum* with trimmed roots, leaving approximately 1 cm of roots to facilitate fungal infection (Lamo et al., 2018). Cut roots were subsequently dipped for 5 min in either a suspension of 10^7^ spores mL^-1^ or autoclaved *F. oxysporum* spores as mock controls (Day 24, **Figure 2**). This higher inoculum concentration with the root-dip method was used for *F. oxysporum* because this soil-borne, vascular wilt pathogen infects plants primarily through the roots and requires successful colonization of the rhizosphere and xylem vessels to confer disease (Constantin et al., 2019; van der Does et al., 2019). Then, each of the challenged plants was instantly potted for three weeks as a challenge period. In total, six treatments (uninoculated control + heat-killed *F. oxysporum*, uninoculated control + *F. oxysporum*, inoculated *K. radicincitans* + heat-killed *F. oxysporum*, inoculated *K. radicincitans* + *F. oxysporum*, inoculated *P. phytofirmans* + heat-killed *F. oxysporum*, and inoculated *P. phytofirmans* + *F. oxysporum*) were tested. Plant growth parameters (i.e., fresh and dry weight, plant height, and number of leaves per plant) and disease symptoms were scored (Day 45, **Figure 2**).

#### 2.4.3 Fusarium disease bioassay

Disease score was determined by counting the number of brown vessels and assessing external growth wilting symptoms, such as yellowing, as described by Constantin et al. (2019). In brief, vascular browning at the cotyledon level was assessed, where 0 = healthy plant with no brown vessels; 1 = brown vessel(s) only at the basal level (above the soil) (below the level of the cotyledons); 2 = one or two brown vessels at the cotyledon level, and the plant still looks healthy; 3 = at least three brown vessels, and the plant shows clear external wilting symptoms (growth distortion) (strong bending of the stem and asymmetric development); 4 = all vessels are brown, and the plant shows clear size reduction (the plant is small and wilted); 5 = the plant is dead.

#### 2.4.4 Fusarium recovery assay

The height of each node on the plant stem was measured before harvesting. Then, plants were harvested, and root and stem pieces were surface sterilized by submergence in 70% ethanol and washed by submergence in sterile tap water. Later, slices of both root and stem nodes were cut with a sterile scalpel, and they were put on a plate made of PDA with supplementary 200 mg L^-1^ of streptomycin and 112 mg L^-1^ penicillin from the antibiotic mix (CELLPURE^®^ Pen/Strep-PreMix, Carl Roth) to reduce bacterial growth. Fungal outgrowth was scored after 48 h of incubation in the dark at 24 °C (Day 47, **Figure 2**). *Fusarium oxysporum* colonization was measured (in cm) when fungal outgrowth was observed at the maximum distance from the soil (van der Does et al., 2019).

### 2.5 Statistical analysis

Statistical analysis was performed with SPSS v.26 (IBM Corp., Armonk, NY, USA). Prior to analysis, all data were assessed for normality (Shapiro–Wilk test) and homogeneity of variance (Levene’s test), with *p* > 0.05 used as the threshold for each assumption. Parametric tests were applied when both assumptions were satisfied; non-parametric alternatives were used otherwise. One-way ANOVA followed by Tukey’s *post hoc* test (*p* < 0.05) was applied to datasets meeting parametric assumptions, including dual culture assays, selected plant growth parameters (e.g., root/shoot weight and shoot length) in greenhouse experiments, lesion diameter, *SlWRKY70* expression, and fungal outgrowth, as this approach controls the family-wise error rate across multiple group comparisons. Where assumptions were violated, the Kruskal-Wallis test followed by Bonferroni correction was applied to two-compartment assays, shoot fresh/dry weight, bacterial quantification, biocontrol efficiency, and most gene expression data, to control Type I error across multiple pairwise comparisons. Leaf count data were analyzed using a generalized linear model (GLM) with Poisson distribution and log link function, appropriate for discrete, non-negative count data. Chi-square tests followed by Z-tests with Bonferroni correction were used to analyze necrosis severity levels. Mann–Whitney U-tests were used for bacterial colonization, disease index, and fungal colonization; the Wilcoxon rank-sum test applied specifically for bacterial colonization in tomato roots, given small sample sizes and non-normal distributions for which rank-based methods are more appropriate. Two-way ANOVA was additionally performed on plant growth and gene expression data to evaluate factor interactions (*p* < 0.05). R software v. 4.2.1 (R Core Team, 2022) was used with the ggstatsplot package (Patil, 2021) to visualize the bacterial colonization of tomato roots and the ggplot2 R package (Wickham, 2016) to obtain all visualized figures.

## 3 Results

### 3.1 Antagonistic assays

**Figure 3:**
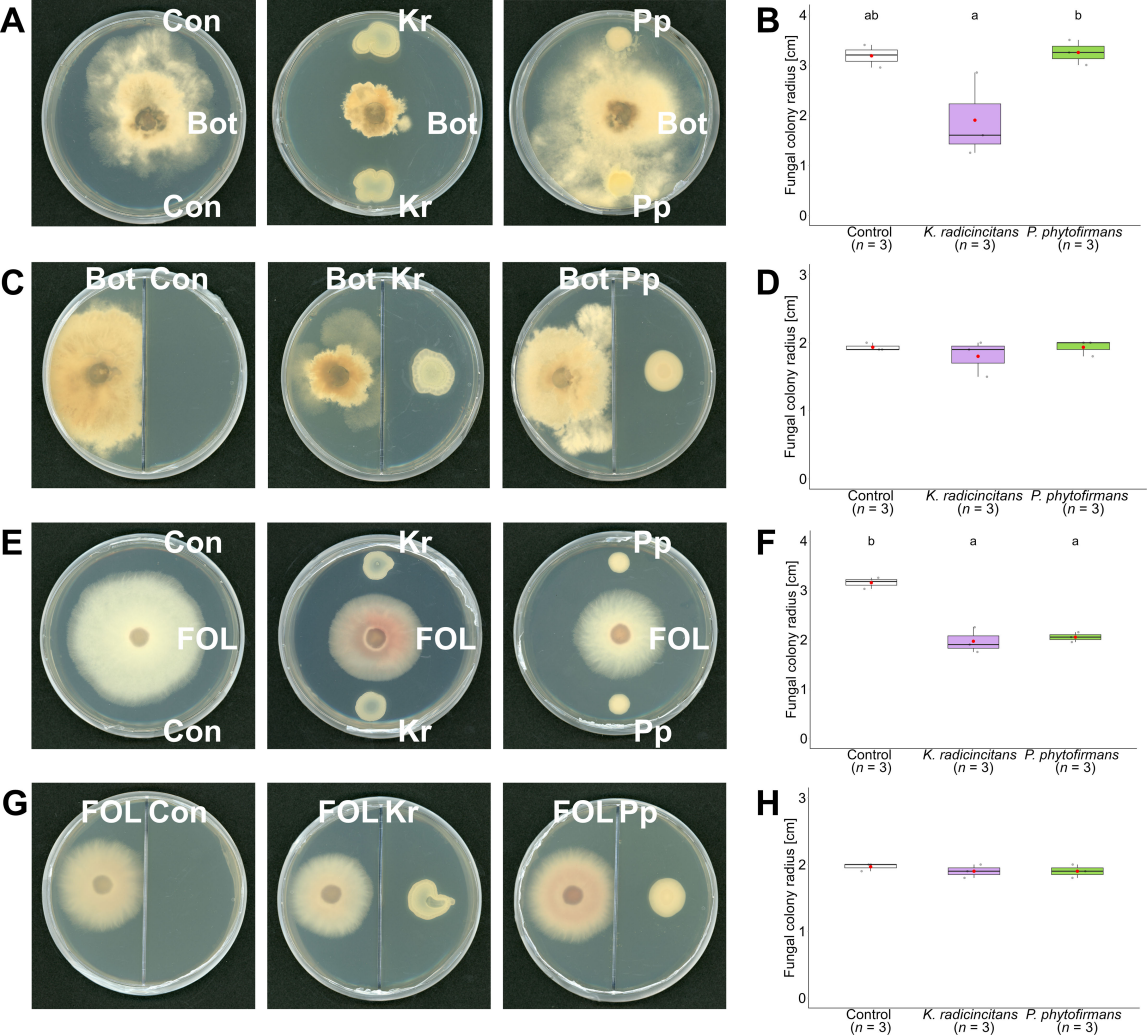
Antagonistic activities of bacterial endophytes against *Botrytis cinerea* and *Fusarium oxysporum* f.sp. *lycopersici* in dual culture and two-chamber plate assays. Growth radii of *B. cinerea* (Bot) and *F. oxysporum* f.sp. *lycopersici* (FOL) were measured in the presence of *Kosakonia radicincitans* (Kr) and *Paraburkholderia phytofirmans* (Pp), with 10 mM MgCl_2_ serving as a negative control (Con). Representative scan images of fungal mycelium are shown for *B. cinerea* in direct dual culture **(A)** and in the two-chamber plate **(C)**, and for FOL under the same respective conditions **(E, G)**. Corresponding quantitative analyses are presented as box plots in panels **(B, D, F, H)** Box plots display the median (solid line), interquartile range (25^th^ – 75^th^ percentiles), raw data points (grey dots), and group means (red dots). Mycelium images were acquired using an Epson Perfection V850 Pro scanner (Epson Scan, v3.9.3.4; 24-bit color, 300 dpi). Statistical differences among treatment means were assessed by one-way ANOVA followed by Tukey's HSD post-hoc test (*n* = 3; *p* < 0.05); when normality or homoscedasticity assumptions were not met, the Kruskal–Wallis test with Mann–Whitney U pairwise comparisons and Bonferroni correction was applied. Groups not sharing a letter differ significantly.

*In vitro* antagonism experiments were performed to measure the growth of phytopathogen fungi with bacterial endophytes by growing the microbes on solid media in Petri dish-based assays in both dual culture and two-compartment plates. Colony radius of *B*. *cinerea* and *F*. *oxysporum* () was recorded after four and seven days of growth on PDA, respectively. In the case of *K. radicincitans*, the effect of diffusible antifungal compounds had significant inhibition of the hyphal growth of both *B. cinerea* (40.3%) (F = 6.83, *p* = 0.028) (**Figures 3A, B**) and *F. oxysporum* (37.6%) (F = 44.13, *p* = 0.0002) (**Figures 3E, F**), with approximately 40% of growth inhibition as well. However, volatile antifungal metabolites of *K. radicincitans* showed a slight inhibition of *B. cinerea* growth (10%) (**Figures 3C, D**) and minor inhibition of *F. oxysporum* growth (2%) (**Figures 3G, H**).

In reference to *P. phytofirmans*, the diffusible antifungal metabolites did not show any significant inhibition effect on the mycelia growth of *B. cinerea* (**Figures 3A, B**), while they significantly inhibited the growth of *F. oxysporum* (34.9%) (F = 44.13, *p* = 0.0002) (**Figures 3E, F**). In addition, the volatile antifungal metabolites of *P. phytofirmans* had no significant difference in inhibition against phytopathogenic fungi (**Figures 3C, D**; **Figures 3G, H**).

### 3.2 Two bacteria confirm their PGPB effects on tomato growth parameters

**Figure 4:**
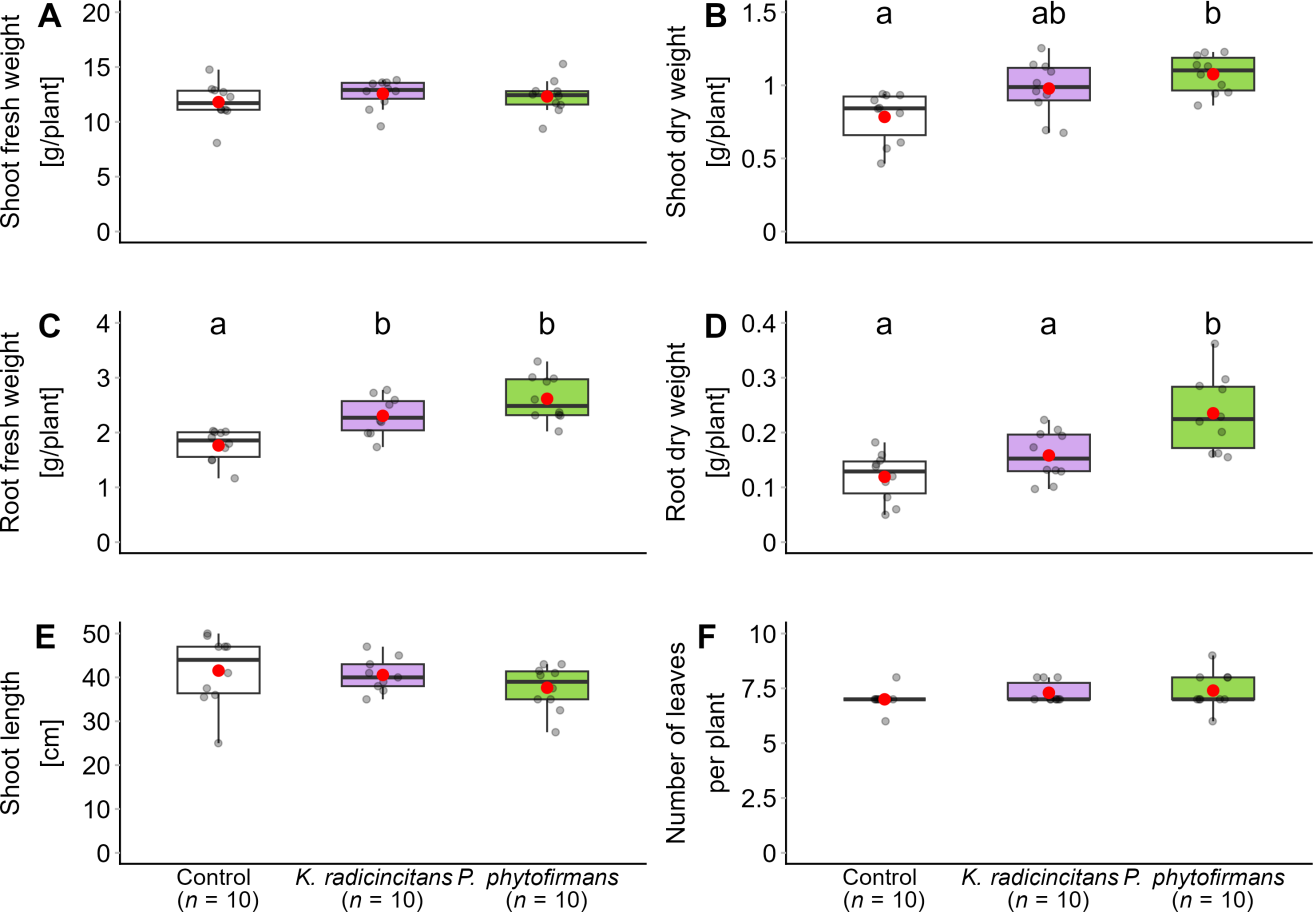
Effect of PGPB, *Kosakonia radicincitans* and *Paraburkholderia phytofirmans*, on tomato growth parameters under greenhouse conditions for the Botrytis experiment. The parameters are **(A)** shoot fresh weight, **(B)** shoot dry weight, **(C)** root fresh weight, **(D)** root dry weight, **(E)** shoot length, and **(F)** number of leaves per plant. Data were recorded one month after bacterial treatments. Box plots show the median (solid line) and the 25^th^ and 75^th^ percentiles; grey dots represent raw data, while red dots represent the mean values. Means not sharing any letters, significantly differ from each other according to the post-hoc Tukey HSD test after one-way analysis of variance (ANOVA) (*p* < 0.05, *n* = 10). If assumptions were violated for data or their natural log transformation, the Kruskal-Wallis test was conducted with the Mann–Whitney U test with Bonferroni correction for multiple comparisons (*p* < 0.05). Moreover, GLM with Poisson distribution was estimated for the number of leaves *(p* < 0.05, *n* = 10) before Bonferroni’s correction.

The two PGPB that showed more than 35% inhibitory effects against *B. cinerea* in dual culture tests were evaluated in greenhouse conditions to test their ability as a potential biocontrol. Before the role of two PGPB in gray mold control was investigated, it was analyzed if plant growth parameters are affected by the two PGPB selected for the present study. The greenhouse experiment involved cultivating the tomato cultivar ‘Moneymaker’ with or without PGPB inoculation at transplanting. The plants underwent normal development and exhibited no symptoms for 30 days. PGPB generally enhanced plant growth compared to the control plants (**Figure 4**). All bacterial treatments showed a statistically significant difference in the shoot dry weight (value = 12.668, *p* = 0.002, Kruskal–Wallis test) (**Figure 4B**). *Paraburkholderia phytofirmans* had significantly higher shoot dry weight than control plants (*p* = 0.002, Mann–Whitney U test with Bonferroni correction). However, there was no significant difference in the shoot dry weight between the *K. radicincitans* inoculated plant or the control plants and between inoculated *K. radicincitans* and *P. phytofirmans* (*p* = 0.051 and *p* = 0.824, respectively, Mann–Whitney U test with Bonferroni correction). Root fresh weights were significantly increased by *K. radicincitans* and *P. phytofirmans* compared to control (F = 14.85, *p* = 0.004 *10^-2^) (**Figure 4C**). Fresh root weight increased by 31% and 49%, respectively, compared to control. Root dry weight was only enhanced by *P. phytofirmans* treatment (F = 11.91, *p* = 0.002 *10^-1^) (**Figure 4D**). There was no significant effect on fresh weight of the shoot (value = 2.248, *p* = 0.325, Kruskal–Wallis test), shoot length (F = 1.343, *p* = 0.280), or number of leaves (*p* = 0.942, GLM) (**Figures 4A, E, F**).

### 3.3 Botrytis bioassay

**Figure 5:**
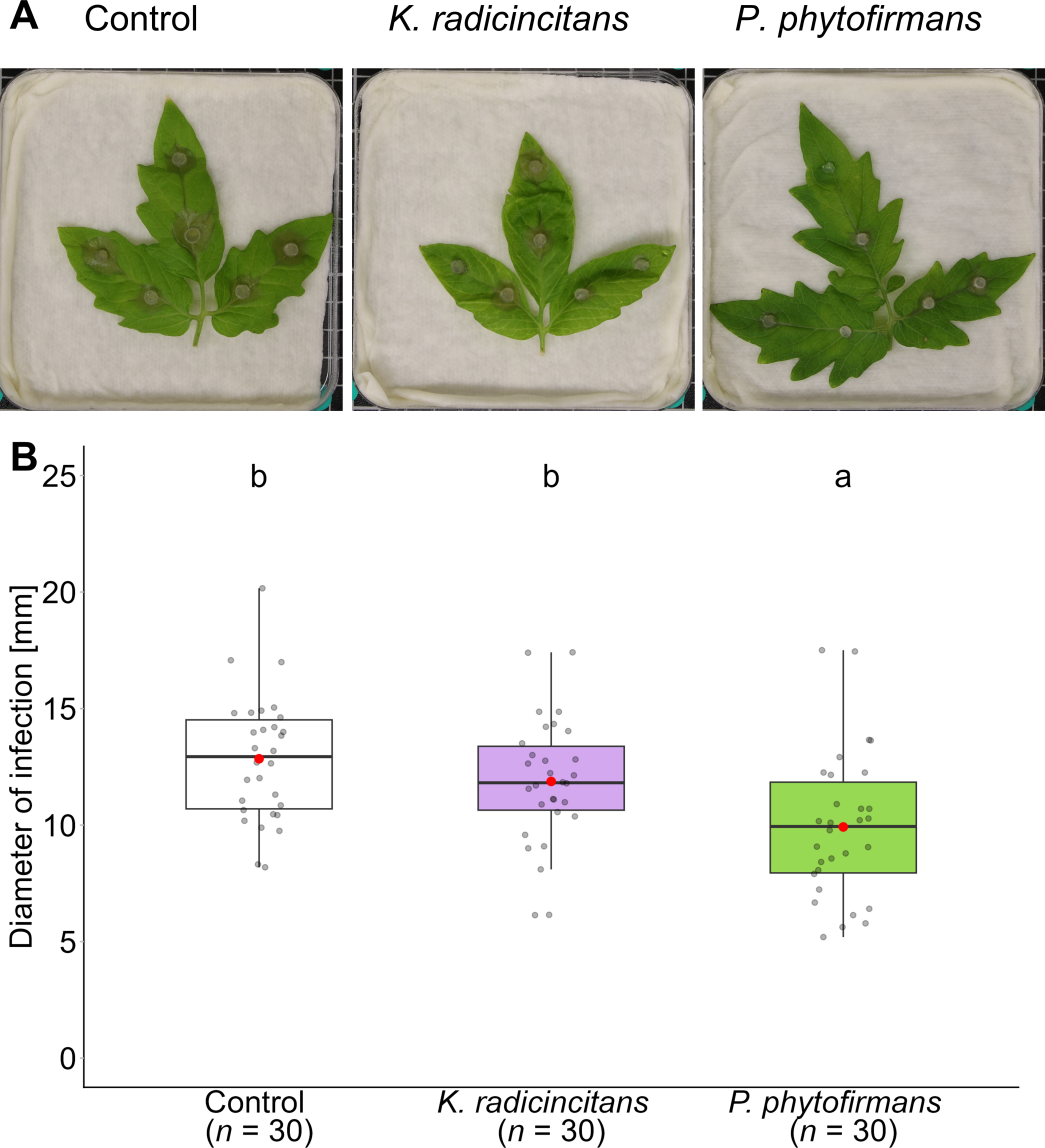
Effect of root inoculation with the plant growth-promoting bacteria *Kosakonia radicincitans* or *Paraburkholderia phytofirmans* on tomato resistance against *Botrytis cinerea* in the tomato cultivar *Solanum lycopersicum*. **(A)** Symptom severity in detached terminal three tomato leaflets of the fourth leaf upon challenge with *B. cinerea* plugs for non-inoculated (control) plants, plants inoculated with *K. radicincitans*, or plants inoculated with *P. phytofirmans*, with **(B)** relative statistical analysis. Disease scoring was determined by measuring the average of the longest and shortest diameters of the necrotic lesions 3 dpi of pathogen inoculation by ImageJ/Fiji v2.10 software. Box plots show the median (solid line) and the 25^th^ and 75^th^ percentiles; grey dots represent raw data, while red dots represent the mean values. Means not sharing any letters, significantly differ from each other according to the post-hoc Tukey HSD test after One-way ANOVA (5 plants, each containing three terminal leaflets, each leaflet having two plugs per leaflet totaling *n* = 30 samples; *p* < 0.05). .

In the same greenhouse experiment, we tested the efficiency of PGPB to increase tomato resistance against *B. cinerea*. Briefly, to assess whether root inoculation is associated with enhanced resistance in distal leaf tissue, the detached three-terminal leaflet of the 4^th^ leaf was challenged by *B. cinerea* plugs and incubated at 21 °C for three days in a climate chamber. *P. phytofirmans* colonization resulted in fewer disease symptoms in the long term (F = 8.19, *p* = 0.001) (**Figures 5A, B**). However, *K. radicincitans* had no significant effect on lesion diameters due to infection. These findings were confirmed by estimating the percentage of biocontrol efficiency (**Supplementary Table 4**).

**Figure 6:**
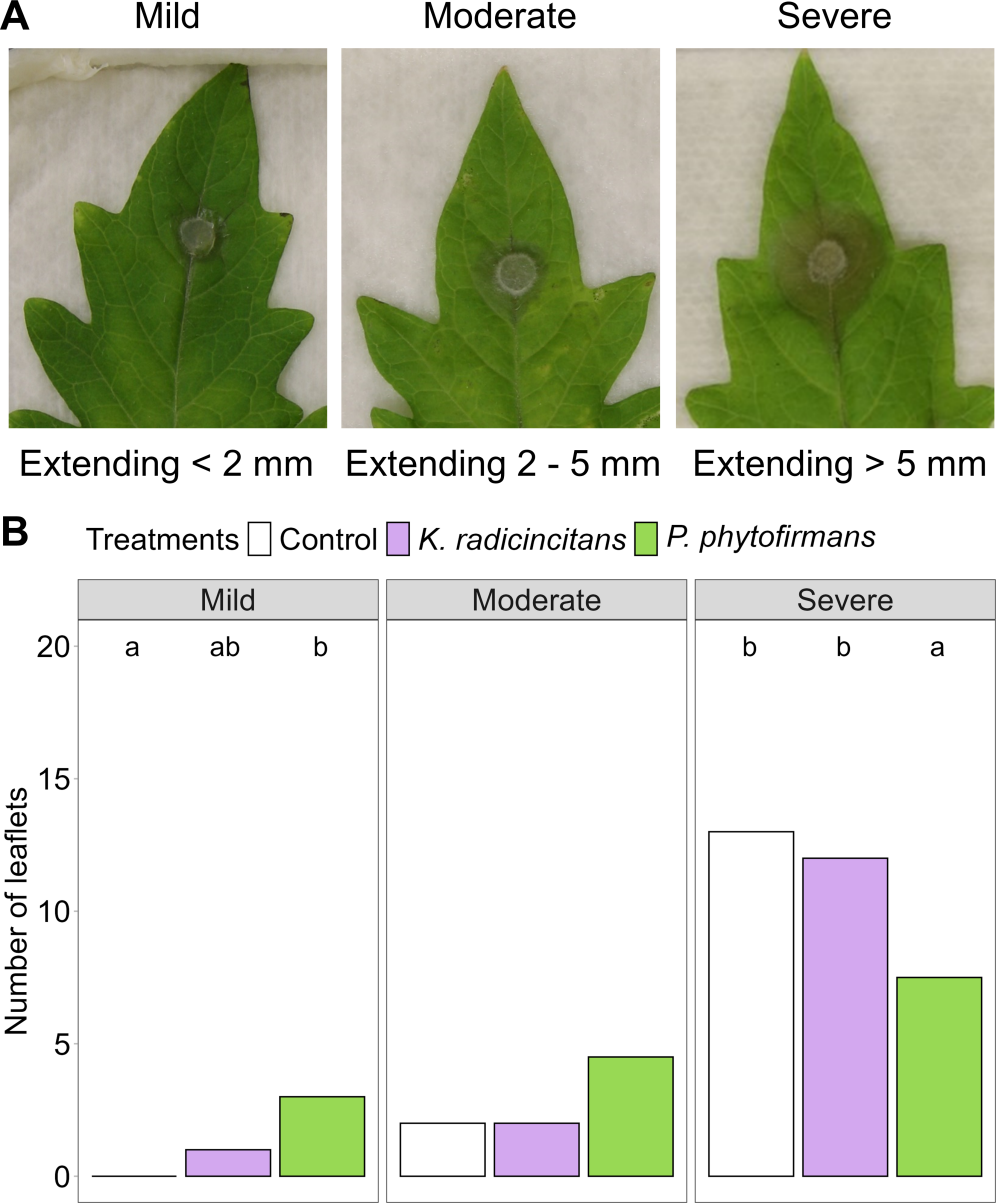
Effect of root inoculation with the two plant growth-promoting bacteria *Kosakonia radicincitans* and *Paraburkholderia phytofirmans* against *Botrytis cinerea* disease development in tomato. **(A)** Necrosis severity of necrosis caused by *B. cinerea* in detached tomato leaflets was scored using three levels of disease scale: mild, moderate, and severe, extending around the plug at < 2 mm, 2 to 5 mm, and > 5 mm, respectively. **(B)** Disease damage level was compared between the control and the two bacterial treatments. Plants were not inoculated as a control or with *K. radicincitans* or *P. phytofirmans* for one month before the challenge with *B. cinerea* plugs. The number of leaflets within each category is shown. Each leaflet had two fungal plugs. Bars not sharing a common letter are significantly different using Bonferroni-corrected Chi-square tests (*χ*^2^), followed by z-tests (*p* < 0.05).

To determine the influence of PGPB on Botrytis disease severity, we recorded a disease index containing three main categories (e.g., mild, moderate, and severe) (**Figure 6A**). Levels of leaf fungal damage were statistically significantly associated with inoculation treatments in the “Moneymaker” cultivar (*X^2^* = 13.11, *p* = 0.011). *Paraburkholderia phytofirmans* protected plants infested with *B. cinerea* plugs by reducing the severity of damage caused by the pathogen, as evidenced by a significant decrease in the number of the most severely damaged leaflets (**Figure 6B**). However, *K. radicincitans*-inoculated plants did not show any significant difference in disease symptoms compared to the control plants.

### 3.4 Colonization of plant tissues by PGPB

In the same greenhouse experiment, the *B. cinerea* pathogen was treated in the leaves, while *K. radicincitans* or *P. phytofirmans* was injected into the soil near the root. This spatial separation of the two microorganisms indicates that protection is associated with the activation of plant defense mechanisms. To test if the exclusion of probable direct antifungal activity of the bacteria was due to the possible colonization of the leaf tissue during the long-term treatments, we investigated the presence of the bacteria in roots and leaves of inoculated and non-inoculated tomato plants using the FdnaJ gene-specific primer pair for *K. radicincitans* and the TaqMan probe Bphyt_1824 gene for the *P. phytofirmans*. Consequently, *K. radicincitans* or *P. phytofirmans* were detected in the roots of the transplanted plants 30 days after bacterial inoculation (Day 44, **Figure 1**). The result indicates that both bacteria successfully colonized the plant’s roots (**Supplementary Figure 3** and **S4**). Moreover, *P. phytofirmans* showed higher colonization than *K. radicincitans* (**Supplementary Figure 3**), while both bacterial strains were not detected in the control plants (data not shown). No bacterial gene amplification was found in the leaves of the same plants. These results were visually confirmed by running agarose gel electrophoresis on the products of their qPCR analysis (**Supplementary Figures 4A, B**).

### 3.5 Expression analysis reveals limited activation of defense pathways

**Figure 7:**
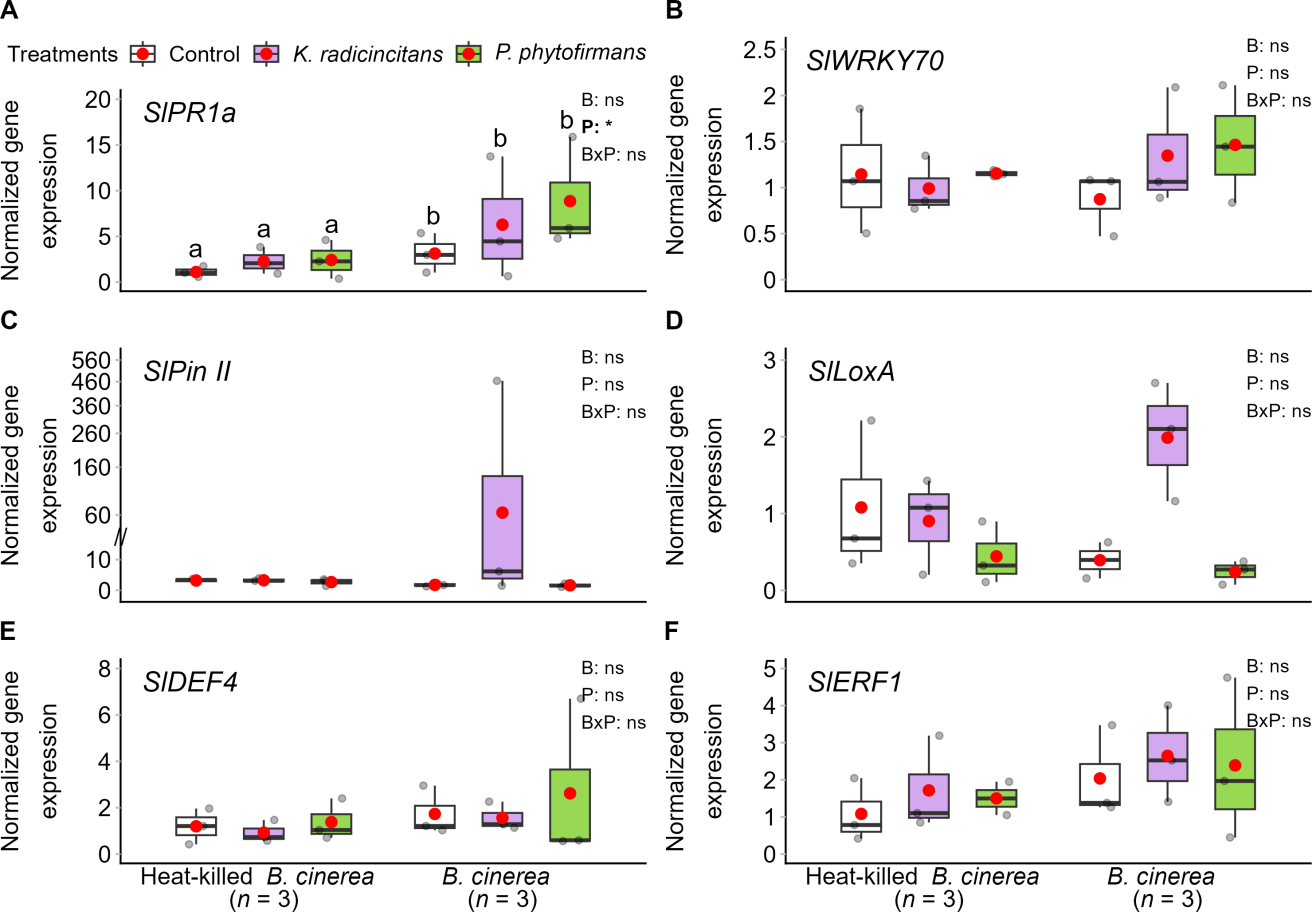
Normalized expression levels of defense-related genes in tomato. Seedlings were not inoculated (control) or inoculated with *Kosakonia radicincitans* or *Paraburkholderia phytofirmans* and challenged after one month with a suspension of *Botrytis cinerea* spores with an autoclaved suspension as heat-killed *B. cinerea* for 3 dpi on detached leaflets. RNA was extracted, and cDNA was synthesized. RNA accumulation of target genes **(A)** *SlPR1a* and **(B)** *SlWRKY70* for the SA pathway, **(C)** *SlPin II* and **(D)** *SlLoxA* for the JA pathway, and **(E)** *SlDEF4* and **(F)** *SlERF1* for the Et-JA pathway were determined by qRT-PCR and normalized with the geometric mean of two reference genes (*SlActin* and *SlGAPDH*). Shown are the calibrated normalized relative quantity (CNRQ) values of all treatments divided by the values of the control treatment (non-inoculated and unchallenged plants). Box plots show the median (solid line) and the 25^th^ and 75^th^ percentiles; grey dots represent raw data, while red dots represent the mean values. One-way ANOVA (*p* < 0.05, *n* = 3) on CNRQ showed no significance between all treatments for all target genes. If ANOVA assumptions were violated, the Kruskal-Wallis test was conducted with Bonferroni correction for multiple comparisons (*p* < 0.05, *n* = 3). Two-way factorial ANOVA was performed using bacterial inoculation **(B)** and pathogen challenge (P) as factors. Each factor's significance values and interactions (B×P) are indicated in the upper-right corner of each graph. Asterisks denote the significant effect of a factor and its interaction. ns: no significant *p* > 0.05 and *: *p* < 0.05. If two-way ANOVA assumptions were violated, the Kruskal-Wallis test was conducted with Bonferroni correction for multiple comparisons for bacterial treatment (*p* < 0.05, *n* = 3) and the Independent-Samples Mann-Whitney U Test (*p* < 0.05, *n* = 3). Means not sharing any letters, significantly differ from each other according to the post-hoc Tukey HSD test after Two-way ANOVA. No other significant impacts were observed. The symbol (//) indicates a scale break on the Y-axis.

To ascertain whether defense signaling pathways were engaged and to comprehend the mechanism behind this long-term induced resistance, we compared the RNA accumulation levels of six selected genes of well-characterized marker genes for SA, JA, and ET signaling pathways in tomato leaves (**Figure 7**). These leaves were obtained from the greenhouse experiment after a challenge with autoclaved *B. cinerea* spore suspension (mock suspension) or *B. cinerea* spore suspension within plants inoculated or not with *K. radicincitans* or *P. phytofirmans* 30 days before the challenge with the pathogen. Gene expression was quantified 72 h after pathogen challenge in detached leaves; therefore, the data do not indicate constitutive activation of defense before excision. Instead, if the expression after infection is elevated, distal leaves exhibit an enhanced defensive response that persists after detachment, consistent with a resistance phenotype preserved in excised tissue. The genes were normalized with geometric means of the best selected reference genes, *SlActin* and *SlGAPDH*, which showed a clear difference of amplicon size between relative DNA and cDNA (**Supplementary Figure 2**). This methodology helps detect any contamination of DNA with cDNA before conducting qPCR assays. The results showed that inoculation with neither of the PGPB impacted the expression of the 6 genes analyzed in challenged or non-challenged plants (**Figure 7**). *Botrytis cinerea* infestation resulted in the upregulation of only the tomato gene encoding acidic pathogenesis-related protein 1 (*SlPR1a*), which was upregulated 4-, 6-, or 8-fold in control, *K. radicincitans*-, or *P. phytofirmans*-colonized plants, respectively (**Figure 7A**). This finding was confirmed by a two-way ANOVA (**Figure 7A**). This enhanced expression of *SlPR1a* in *P. phytofirmans*-colonized plants in response to pathogen challenge is suggestive of an ISR-like response; however, this single time-point observation is insufficient to confirm a primed state. Priming requires demonstration of faster or stronger induction kinetics relative to non-primed controls (Conrath et al., 2006; Pieterse et al., 2014), which our experimental design does not provide. This finding therefore remains consistent with, but does not confirm, a possible priming-associated mechanism. No significant interaction between *B. cinerea* and PGPB colonization was observed for the gene *SlWRKY70* transcription factor in tomato (**Figure 7B**). JA pathway-regulated genes encoding proteinase inhibitor II (*SlPin II*) (**Figure 7C**) and JA-inducible lipoxygenase (*SlLoxA*) (**Figure 7D**) did not show a significant difference in their expression between all treatments. In the presence of *B. cinerea*, inoculated plants or those not inoculated with *P. phytofirmans* showed a very low level of expression of both JA-regulated genes. Plants colonized by *K. radicincitans* upregulated *SlPin II* and *SlLoxA* upon *B. cinerea* infestation (**Figures 7C, D**). However, *B. cinerea* induced disease in the presence of *K. radicincitans*-bacterized plants (**Figures 6A, B**). Regarding the ET-regulated signaling pathway reflected by the expression of genes encoding defensin-like proteins 4 (*SlDEF4*) (**Figure 7E**) and ethylene response factor 1 (*SlERF1*) (**Figure 7F**), no significant difference was observed among all treatments.

### 3.6 Impact of PGPB and Fusarium on tomato vegetative growth

To analyze the effects of the two PGPB and *F. oxysporum* on tomato growth, tomato seedlings were inoculated with *K. radicincitans* or *P. phytofirmans* or mock-inoculated two weeks after sowing. Plants were then challenged with spores of the pathogen by root-dip inoculation for another ten days and harvested to estimate plant growth parameters after a further three weeks of cultivation (**Figure 2**).

**Figure 8:**
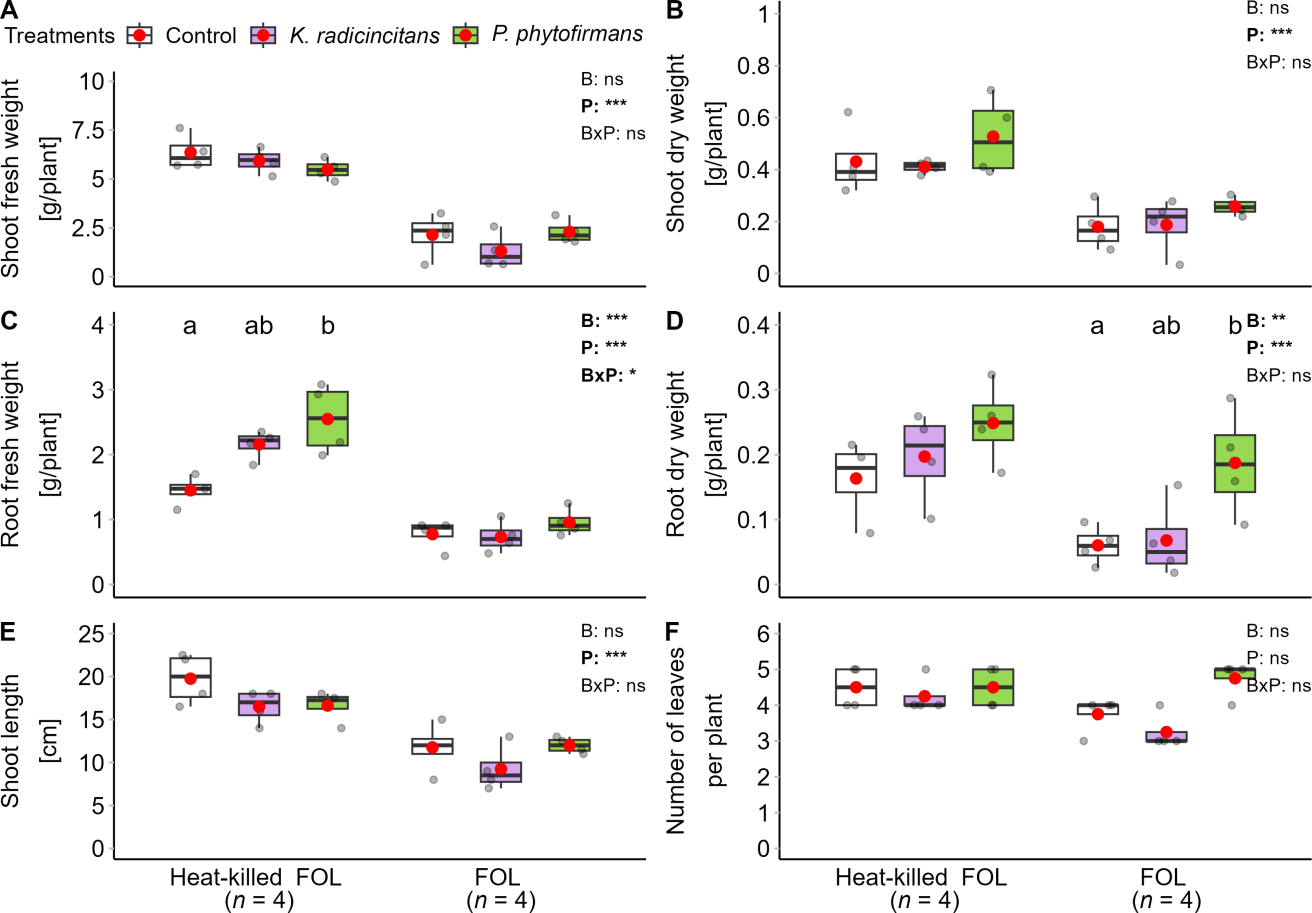
Effect of PGPB, *Kosakonia radicincitans* and *Paraburkholderia phytofirmans*, on tomato growth parameters under greenhouse conditions for *Fusarium oxysporum* f.sp. *lycopersici* (FOL) experiment. The parameters are **(A)** shoot fresh weight, **(B)** shoot dry weight, **(C)** root fresh weight, **(D)** root dry weight, **(E)** shoot length, and **(F)** number of leaves per plant. Data were recorded 21 days after bacterial treatment (11 days of *F. oxysporum* challenge or heat-killed FOL as a mock). Box plots show the median (solid line) and the 25^th^ and 75^th^ percentiles; grey dots represent raw data, while red dots represent the mean values. Two-way factorial ANOVA was performed using bacterial inoculation **(B)** and pathogen challenge (P) as factors. Each factor's significance values and interactions (B×P) are indicated in the upper-right corner of each graph. Asterisks denote the significant effect of a factor and its interaction, ns: no significance, *p* > 0.05 and *: *p* < 0.05, **: *p* < 0.01, and ***: *p* < 0.001. If two-way ANOVA assumptions were violated, the Kruskal-Wallis test was conducted. If the bacterial effect was significant, One-way ANOVA (*p* < 0.05, *n* = 4) was conducted separately in the challenge treatments followed by post-hoc Tukey HSD test for multiple comparisons. If ANOVA assumptions were violated, the Kruskal-Wallis test was conducted with Bonferroni correction for multiple comparisons (*p* < 0.05, *n* = 4) and the Independent-Samples Mann-Whitney U Test (*p* < 0.05, *n* = 4). Means not sharing any letters, significantly differ from each other. If no letters are shown, it indicates no significance was observed.

*Paraburkholderia phytofirmans* significantly increased root fresh weight only in the absence of the *F. oxysporum* challenge (**Figure 8C**) and root dry weight in the presence of the *F. oxysporum* challenge (**Figure 8D**). However, the differences in the other plant growth parameters were rather small, regardless of whether the *F. oxysporum* challenge was absent or present (**Figures 8A, B, E, F**). *Kosakonia radicincitans* did not show any improvement on plant growth parameters (**Figure 8**). Plants challenged with the *F. oxysporum* pathogen were stunted and revealed much lower biomass parameters than non-infected plants regardless of whether the plants were bacterized by *K. radicincitans* or *P. phytofirmans* or not (**Figures 8A–E**). These infected plants increased total dry weight and shoot and root dry weight by 8% and 87% when colonized with *K. radicincitans* or *P. phytofirmans*, respectively, compared to non-bacterized plants. Neither bacterial treatments nor the *F. oxysporum* challenge had a significant effect on the number of leaves (**Figure 8F**). A slight improvement of pathogen-challenged plants by *P. phytofirmans* in disease severity (4) and biocontrol efficiency (5%) were observed, but this improvement was not significant (**Supplementary Table 5**).

### 3.7 No suppression of wilt disease by PGPB

To verify if PGPB can also promote resistance in tomato plants against wilt disease, we estimated the fresh weight and severity of disease symptoms after 21 days of challenging (**Figure 2**). These symptoms were scored with the disease index (DI), ranging from 0 (healthy) to 5 (dead).

**Figure 9:**
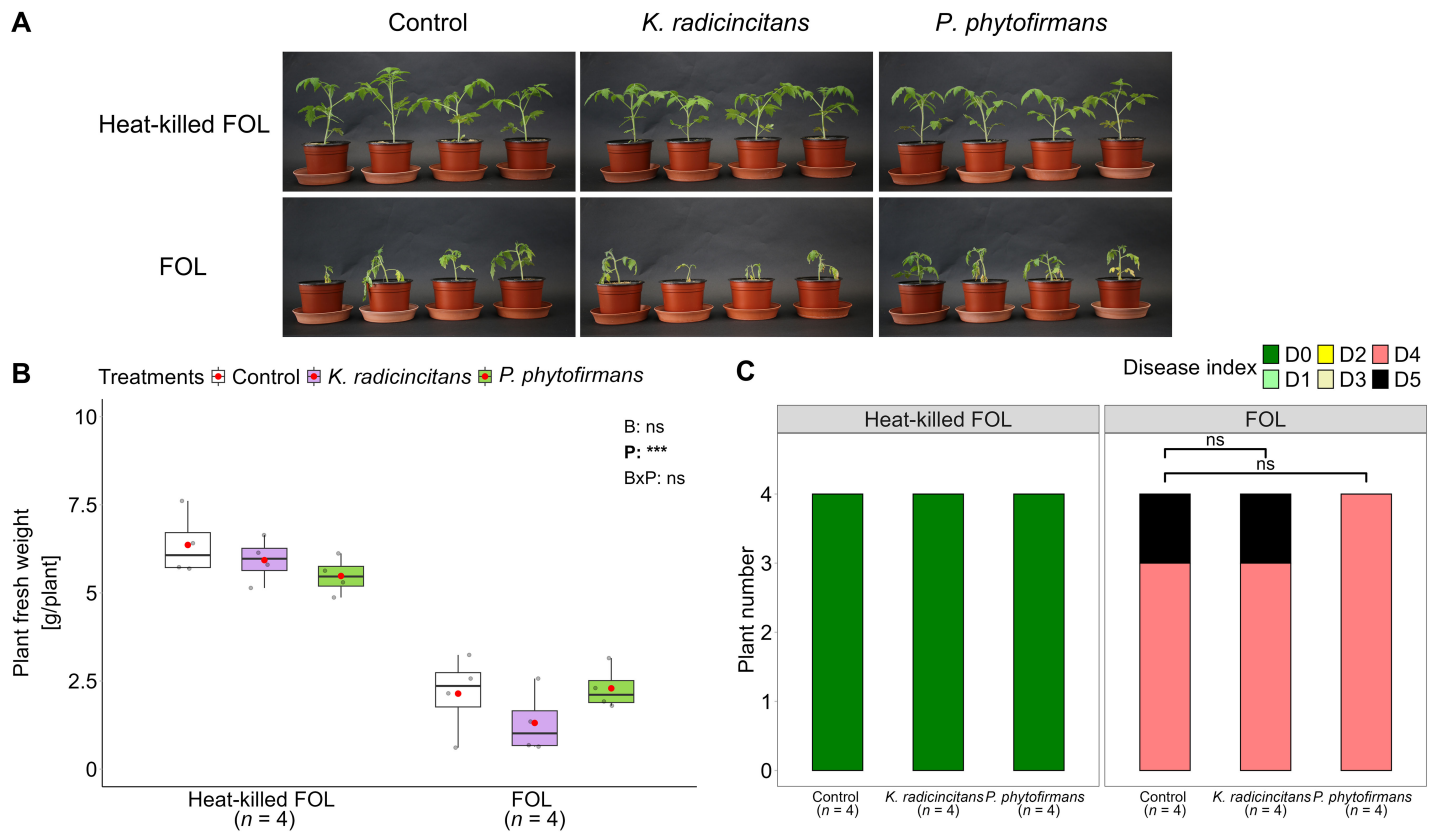
*Kosakonia radicincitans* and *Paraburkholderia phytofirmans* cannot suppress Fusarium wilt disease in tomato plants under greenhouse conditions. **(A)** Tomato plants three weeks after *Fusarium oxysporum* f.sp. *lycopersici* (FOL) pathogen challenge. 24-day-old tomato plants, inoculated with bacterial suspensions or with a MgCl_2_ buffer as a control, were infected two weeks later with a *F. oxysporum* spore suspension (10^7^ CFU mL^-1^) of either autoclaved spore (heat-killed FOL) or *F. oxysporum* suspension and evaluated at 21 days post-infection. Disease symptoms on plants were scored by measuring **(B)** plant fresh weight by determining the weight of the whole plant, including shoot and root parts, of four plants per treatment (*n* = 4). Box plots show the median (solid line) and the 25^th^ and 75^th^ percentiles; grey dots represent raw data, while red dots represent the mean values. Two-way factorial ANOVA was performed using bacterial inoculation **(B)** and pathogen challenge (P) as factors. Each factor's significance values and interactions (B×P) are indicated in the upper-right corner of the graph. Asterisks denote the significant effect of a factor and its interaction, ns: no significance, *p* > 0.05 and ***: *p* < 0.001. **(C)** disease index (ranging from 0 to 5) measured for four plants per treatment. Disease index (DI) = 0: no brown vessels, DI = 1: brown vessel(s) only at the basal level, DI = 2: one or two brown vessels at the cotyledon level, DI = 3: three brown vessels at the cotyledon level, DI = 4: all vessels are brown; DI = 5: the plant is dead. Disease index was analyzed by a non-parametric Mann–Whitney U-test (ns: no significant *p* > 0.05, *n* = 4) compared to its control.

Plant fresh weight did not statistically differ between bacterized and non-bacterized plants either in the absence or the existence of the *F. oxysporum* challenge (**Figures 9A, B**). However, *F. oxysporum* drastically reduced the weight among the challenged plants, including bacterial treatments, compared to unchallenged treatments (**Figure 9B**). Inoculation of *P. phytofirmans* with *F. oxysporum* slightly increased plant fresh weight compared with solely *F. oxysporum*-infected tomato by 11%. Inoculation of *K. radicincitans* with *F. oxysporum* lowered fresh weight by 30% compared to solely *Fusarium*-infested tomato plants (**Figure 9B**). Similarly, tomato plants inoculated with *P. phytofirmans*, and *F. oxysporum* visually reduced wilt disease symptoms (DI = 4) compared with those inoculated with *K. radicincitans* and *F. oxysporum* or plants only infested with *F. oxysporum* (DI = 4~5), but this decrease was not statistical (**Figure 9C**). In addition, plants were either dead or wilted.

### 3.8 PGPB did not limit Fusarium colonization in tomato roots and stems

To better understand how *F. oxysporum* can colonize the plant and how PGPB can limit the spread of the pathogen, a fungal recovery assay was conducted. *Fusarium oxysporum* was reisolated from xylem vessels of roots and stems after three weeks of challenge. The height of nodes on tomato stems was meticulously measured. Roots and stems were then harvested and surface sterilized. Subsequently, a slice of each root and node of stem was harvested and placed on PDA treated with antibiotics in Petri dishes (**Figure 2**). After three days of incubation at 25 °C in the dark, the growth of fungal mycelia was recorded and analyzed.

**Figure 10:**
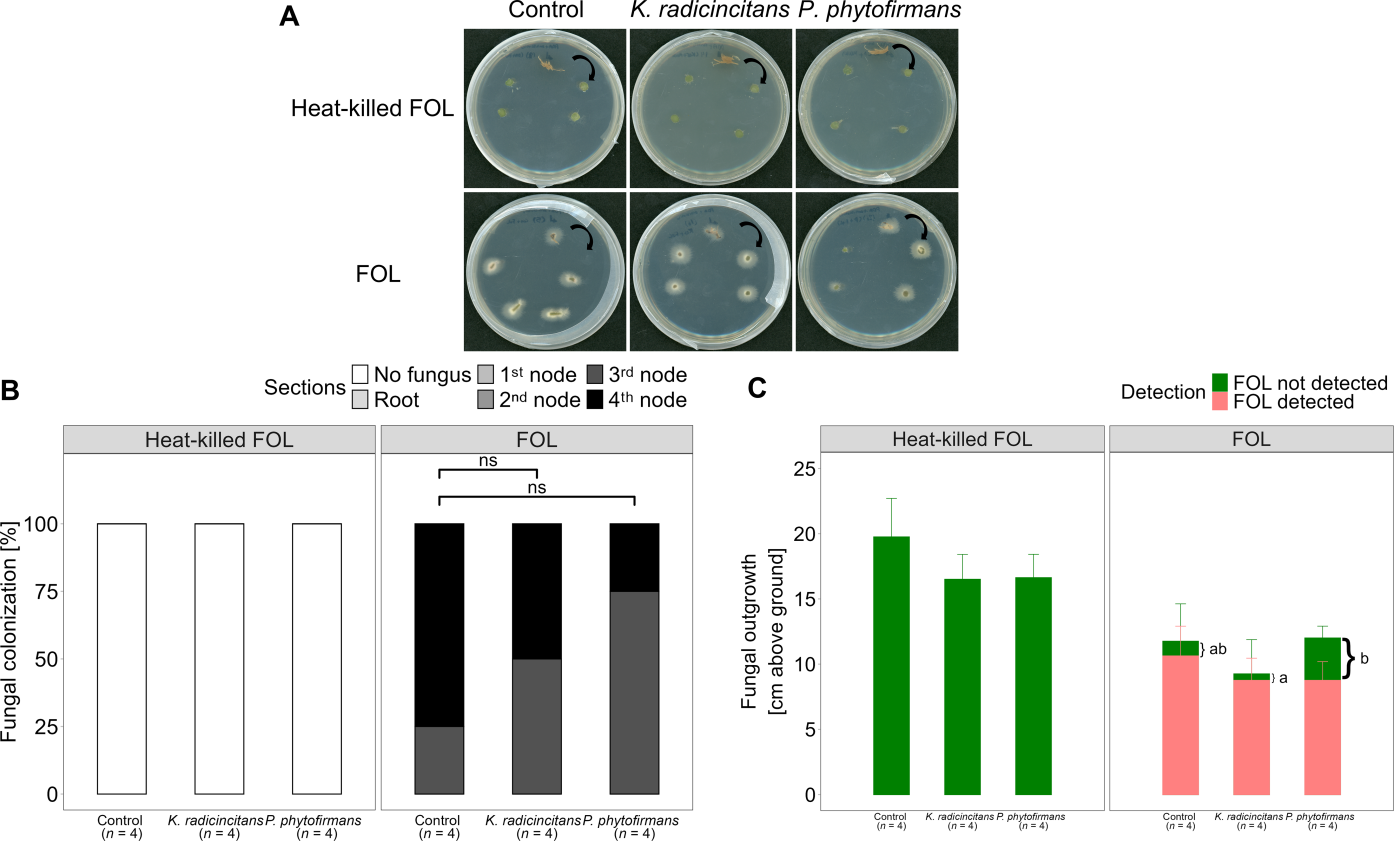
*Fusarium oxysporum* f.sp. *lycopersici* (FOL) recovery assay. 2-week seedlings were inoculated with two plant growth-promoting bacteria, *Kosakonia radicincitans* or *Paraburkholderia phytofirmans* (10^8^ CFU mL^-1^), or with a MgCl_2_ buffer as a control under greenhouse conditions. After 10 days, seedlings were infected with *F. oxysporum* by the root dip method in *F. oxysporum* spore suspension (10^7^ CFU mL^-1^) or autoclaved spore (heat-killed FOL). Tomato plants three weeks after *F. oxysporum* pathogen challenge by the root dip method and evaluated at 21 days post-infection. Then, slices of roots and all stem node pieces (1^st^ node, 2^nd^ node, 3^rd^ node, and 4^th^ node) were surface sterilized, put on a PDA plate, and after incubation for two to three days at 25 °C, fungal outgrowths were scored. **(A)**: Representative plant pieces were taken from the root (top middle of the plate), moving clockwise to the right at the 1^st^ node, 2^nd^ node, 3^rd^ node, and 4^th^ node levels of four individual plants incubated for three days on PDA plates. **(B)** Percentage of *F. oxysporum* colonization (*n* = 4, per treatment). Raw data were analyzed by a non-parametric Mann–Whitney U-test (ns: no significant *p* > 0.05). **(C)** Determination of the height of *F. oxysporum* colonization inside the tomato plant. The distance between each sample and the soil surface was measured in centimetres. Fungal colonization levels were determined as the maximum distance from the soil until fungal outgrowth was detected (in cm). Green bars indicate the differences in plant height between heat-killed *F. oxysporum* plants and *F. oxysporum* -not-detected plants across bacterial treatments while pink bars indicates the differences in the height of *F. oxysporum*-infected plants among bacterial treatments. Black brackets indicates the differences in plant height between *F. oxysporum*-detected and *F. oxysporum*-not-detected plants (measured in cm). Bars indicate the averages of four individual plants. Error bars indicate standard deviation (+SD). Bars not sharing a common letter are significantly at the significance level (5%), according to the Tuckey HSD test using one-way ANOVA (*p* < 0.05).

In all cases, the pathogen reached the fourth node, and the fungus was observed at the third node or below (**Figures 10A, B**). Note that fungal colonization in plants colonized with *P. phytofirmans* only reached 25% of the fourth node, but no significant differences could be observed among all treatments (**Figure 10B**). Control plants without endophytic bacteria were colonized by *F. oxysporum* almost completely (≥ 90%). *Fusarium oxysporum* colonized the plants protected by *P. phytofirmans* up to three-quarters of their height (**Figure 10C**). Relative pathogen spread was lower in *P. phytofirmans* (73% of the total height) than in *K. radicincitans* plants (96%) compared to the positive control (90%) (**Figure 10C**). Interestingly, the distance between the maximum plant height and the highest point of *F. oxysporum* detection was significantly greater in plants protected by *P. phytofirmans* compared to those treated with *K. radicincitans* or control after *F. oxysporum* challenge (**Figure 10C**).

## 4 Discussion

To control diseases in an environmentally friendly way, searching for bioinoculants is an important target for our health. *Kosakonia radicincitans* and *Paraburkholderia phytofirmans*, presented here, are PGPB. This study evaluated the efficacy of *K. radicincitans* and *P. phytofirmans* as PGPB and biocontrol agents against both the foliar necrotrophic pathogen *Botrytis cinerea* and the hemibiotrophic root-infecting fungal pathogen *Fusarium oxysporum* in tomato. Their plant growth promotion activities were evaluated by biomass enhancement and inoculation effectiveness. Direct pathogen antagonism and indirect system resistance were assessed to determine their biocontrol potential. The two PGPB proved markedly different in their performance across the contrasting pathosystems examined.

### 4.1 *In vitro* experiment

To estimate the direct antagonistic effect of tested bacteria against pathogens, we used two different *in vitro* methods: dual culture and two-compartment plates. The inhibition zones could be due to the presence of secreted inhibitory compounds like toxic metabolites, antibiotics, or siderophores (Swadling and Jeffries, 1996). In this study, *K. radicincitans* inhibited the mycelial growth of *B. cinerea* and *F. oxysporum* under *in vitro* conditions, however; this outcome cannot be directly extrapolated to *in planta* performance. The production of antifungal metabolites in dual culture assays occurs under highly controlled, nutrient-rich conditions that may not reflect the regulatory environment of the rhizosphere or plant tissue (Besset-Manzoni et al., 2019; Li et al., 2019). Whether *K. radicincitans* produces comparable metabolites at effective concentrations *in planta* remains unresolved and would require targeted colonization and metabolomics studies to address. This result agrees with the results of Lambrese et al. (2018) which showed inhibition of *B. cinerea* and the results of (Ibrahim et al., 2020) against the soil fungus *Fusarium solani*. In our study, *P. phytofirmans* suppressed only *F. oxysporum* in dual culture, which agrees with results obtained by Mahdikhani and Davoodi (2016) which found that many *Burkholderia* species inhibit *F. oxysporum* f. sp. *melonis in vitro*. However, *P. phytofirmans* did not suppress the growth of *B. cinerea*. This observation aligns with Miotto-Vilanova et al. (2022), which showed that *P. phytofirmans* cannot inhibit *B. cinerea*. However, previous studies showed a clear inhibition of *B. cinerea* (Ait Barka et al., 2000, 2002). The discrepancy may relate to differences in growth medium: our study used King’s B medium, whereas Ait Barka et al. (2000) used PDA, potentially affecting the bacteria’s ability to produce antifungal compounds. *Paraburkholderia phytofirmans* produce multiple metabolites, including antifungal compounds (Partida‐Martinez and Hertweck, 2007; Vial et al., 2007; Schmidt et al., 2009) capable of suppressing a broad spectrum of fungal phytopathogens (Quan et al., 2006; Kilani-Feki and Jaoua, 2011; Groenhagen et al., 2013). The selective inhibition of *F. oxysporum* but not *B. cinerea* may imply that the responses to specific antibiotic substances produced by this strain differ between the two pathogens. Further research is required to clarify the mechanisms of direct antagonistic effects. In the two-compartment plate conditions, neither *K. radicincitans* nor *P. phytofirmans* inhibited *B. cinerea* or *F. oxysporum*. This result is in accordance with Miotto-Vilanova et al. (2022) who showed that *P. phytofirmans* cannot inhibit the mycelial growth of *B. cinerea* via volatile organic compounds, suggesting direct cell contact as the primary mechanism. Our findings further suggest that diffusible bacterial compounds play the main role in the antagonism, while volatile bacterial compounds may be of minor or no importance.

### 4.2 Botrytis greenhouse experiment

Treatment of tomato roots with *P. phytofirmans* reduced disease symptoms upon inoculation with the necrotrophic pathogen *B. cinerea* in the “Moneymaker” cultivar. Neither bacterium was detected in shoot tissues (**Supplementary Figure 4**), indicating physical separation and suggesting that root inoculation by *P. phytofirmans* is associated with enhanced resistance in distal leaf tissue, possibly involving plant-mediated systemic responses, rather than direct antagonism.

Previous studies have confirmed the ability of *P. phytofirmans* to elicit such responses against this necrotroph in *A. thaliana* (Timmermann et al., 2019) and grapevine (Miotto-Vilanova et al., 2016), even without bacterial presence in aerial tissues.

*Kosakonia radicincitans* and *P. phytofirmans* have displayed enhancements in plant nutrition and growth parameters in multiple plant species (Compant et al., 2019; Rojas-Rojas et al., 2025). Improved plant growth plays a role in bioprotection against pathogens through beneficial microorganisms (Whipps, 2001). To evaluate the role of plant growth promotion, we analyzed the bacterial influence on plant biomass parameters. An increase in plant growth induced by *P. phytofirmans* was observed (**Figure 4**) while *K. radicincitans* had no significant effect. This difference may be due to their relative internal root tissue colonization levels (**Supplementary Figure 3**). These findings indicate that the protective effect observed in *P. phytofirmans-*colonized plants may involve both plant growth and defense.

#### 4.2.1 Plant defense

*Kosakonia radicincitans* antagonized the *B. cinerea* mycelia *in vitro*, but it did not control disease development on the plant, while *P. phytofirmans* was *vice versa. Paraburkholderia phytofirmans* inoculation significantly reduced disease symptoms of *B. cinerea* in *S. lycopersicum* cv. “Moneymaker” compared to both *K. radicincitans* and pathogen-only controls (**Figures 6A, B**). Given that bacteria were undetected in challenged leaves (**Supplementary Figures 4A, B**), direct antagonism was unlikely to account for disease reduction. Instead, plant-mediated systemic responses may contribute to disease reduction, though this requires experimental validation. The detached-leaf assay captures a resistance phenotype retained in excised tissue following root colonization, rather than monitoring active systemic signaling (Martínez-Medina et al., 2013; Martínez-Hidalgo et al., 2015). To investigate this hypothesis, we estimated the transcriptional levels of marker genes for the three main signaling pathways involving SA, JA, or ET.

##### 4.2.1.1 SA pathway

The *SlPR1a* and *SlWRKY70* are studied in plant response to biotic and abiotic stresses as markers of SA systemic acquired resistance (SAR) (Zhang et al., 2010; Hu et al., 2012; Mishra et al., 2024; Wang et al., 2025). Neither *P. phytofirmans* nor *K. radicincitans* showed any impact on the expression of *SlPR1a* and *SlWRKY70* in the absence of *B. cinerea* (**Figures 7A,B**). In the presence of *B. cinerea*, however, a significant increase in *SlPR1a* expression was observed in *P. phytofirmans*-colonized plants compared to *K. radicincitans* inoculated plants and non-inoculated control, respectively. *PRa1* proteins have antifungal activity and have been linked to *P. phytofirmans*-mediated resistance in grapevine (Miotto-Vilanova et al., 2016) and other hosts (Gu et al., 2023). Our data suggest that PR proteins may be involved in *P. phytofirmans*-mediated plant resistance to *B. cinerea* disease in tomato plants.

Priming, the enhanced readiness to mount defense responses upon pathogen challenge, is associated with both SAR and ISR (Conrath et al., 2006; Timofeeva et al., 2023). Our data showed that SlPR1a was only induced when Botrytis was present in *P. phytofirmans*-inoculated plants, a pattern consistent with priming. However, as gene expression was assessed at a single time point (72 h post-challenge), we cannot distinguish whether this reflects priming-associated amplification of the defense response, a difference in infection progression between treatments, or differential assay sensitivity. Definitive evidence of priming would require time-course analyses demonstrating faster or stronger induction kinetics upon challenge (Conrath et al., 2006). These findings are consistent with those of Dong-Dong et al. (2011) who showed that SlPR1a is primed in plants treated with *Bacillus cereus* AR156AR upon attack by *Pseudomonas syringae*. Similarly, inoculation with *B. thuringiensis* (Yoshida et al., 2019) and *Peribacillus frigoritolerans* (Mazuecos-Aguilera et al., 2025) suppressed *B. cinerea* in tomato, the latter linked to increased *PR1* gene expression.

##### 4.2.1.2 JA pathway

JA-defense responses usually control necrotrophic pathogens often in synergy with ethylene (Glazebrook, 2005; Stroud et al., 2022) and are crucial to basal resistance against *B. cinerea* in tomatoes (AbuQamar et al., 2008; Zhao et al., 2023). Notably, *B. cinerea* can exploit the SA pathway to suppress JA-mediated defenses (El Oirdi et al., 2011; Martínez-Hidalgo et al., 2015). We evaluated the JA signaling pathway via marker genes *SlPin II* and *SlLoxA*. The results showed no significant difference for both genes across treatments (**Figures 7C,D**). This suggests that the classical ISR involving the JA pathway is not prominently involved in the resistance against *B. cinerea* conferred by *P. phytofirmans*. *Kosakonia radicincitans* showed moderate upregulation of SA–JA pathway markers upon Botrytis-related necrosis (**Figure 5**; **Figures 7A, C, D**), possibly reflecting SA–JA crosstalk under pathogen pressure (Mur et al., 2006).

##### 4.2.1.3 ET-JA mediated pathway

ET modulates crosstalk between SA and JA and, together with JA, contributes to resistance against necrotrophic fungi (Kunkel and Brooks, 2002; Ali et al., 2025). To estimate the role of the ET pathway, we estimated the transcription of the *SlERF1* gene encoding the ET-responsive transcription factor 1 and *SlDEF4* gene encoding defensin-like proteins 4 (Zarei et al., 2011; Ding et al., 2022). Neither gene differed significantly across treatments with or without pathogen challenge. Thus, ET-JA-mediated signaling does not appear to contribute substantially to the protection observed here.

### 4.3 Fusarium greenhouse experiment

Several PGPB taxa, including Bacillus, Streptomyces, and Paenibacillus, have been reported to suppress Fusarium wilt in tomato through antibiotic production, competition, and induction of SA-mediated defenses (Abbasi et al., 2019; Syed Nabi et al., 2021; Ansari et al., 2024).

In the Fusarium greenhouse experiment, neither *K. radicincitans* nor *P. phytofirmans* significantly suppressed the vascular wilt disease in the tomato cultivar “Moneymaker,” which is susceptible to *F. oxysporum* race 3 (Catanzariti et al., 2015). *Paraburkholderia phytofirmans* slightly reduced disease symptoms and enhanced root dry weight relative to *K. radicincitans* (**Figures 8D**; **9**), but re-isolation of *F. oxysporum* from tomato stem nodes revealed that *P. phytofirmans* did not significantly lower the extent of pathogen spread (**Figure 10**). Although *K. radicincitans* and *P. phytofirmans* antagonize *F. oxysporum in vitro*, these mechanisms were insufficient to restrict infection under greenhouse conditions. The root-dip inoculation method combined with inoculum density of 10^7^ spores mL^−1^ represented a stringent test of biocontrol efficacy (Constantin et al., 2019; van der Does et al., 2019). This high disease pressure may have masked more subtle protective effects under lower inoculum densities.

Although *K. radicincitans* and *P. phytofirmans* displayed antagonistic activity against *F. oxysporum in vitro*, this did not translate into significant disease suppression. Possible contributing factors include reduced rhizosphere persistence, altered metabolite production in soil, or competitive exclusion by native microbiota. This outcome is consistent with the well-documented observation that *in vitro* screening of biocontrol candidates is a poor predictor of in planta efficacy (Pliego et al., 2011; Gómez Expósito et al., 2017), underscoring the importance of rhizosphere competence evaluation in biocontrol candidate selection. Successful plant protection did not follow from *in vitro* antagonism, consistent with Besset Besset-Manzoni et al. (2019), and may reflect competitive exclusion or altered metabolite production in soil (Knudsen et al., 1997). Earlier, Frommel et al. (1991b) showed that co-inoculation of *P. phytofirmans* with *Serratia plymuthica* elevated resistance against *F. oxysporum* disease in tomato plants; however, our study did not replicate this finding. Together, these results *F. oxysporum* infection pressure under the conditions tested exceeded the biocontrol capacity of both strains.

### 4.4 Limitation

*Kosakonia radicincitans* did not significantly improve biomass (**Figure 8**) or confer protection against Botrytis or Fusarium in the *in planta*, despite antagonizing both fungal pathogens *in vitro* (**Figure 3**). An explanation for the inadequate performance of *K. radicincitans* may be that The inoculum was prepared from lyophilized powder stored at -80 °C for 12 months, which may have impaired plant growth-promoting (PGP) functionality. Berger et al. (2018) observed beneficial effects with a 6-month-old solid inoculum stored at −20 °C, suggesting that storage conditions may be critical.

Cell viability, as measured by colony-forming units, does not always reflect functional competence of plant growth-promoting (PGP) traits. Lyophilization and long-term storage can inflict sub-lethal damage on non-sporulating Gram-negative bacteria, impairing metabolically demanding functions such as nitrogen fixation, auxin biosynthesis, siderophore production, and phosphate solubilization (Martínez-Viveros et al., 2010; Berninger et al., 2018). Although *K. radicincitans* encodes multiple PGP-relevant genes (Bergottini et al., 2015), extended storage at −80 °C for 12 months may have reduced their expression or activity without affecting CFU counts. The lack of in planta effects may therefore reflect storage-induced impairment rather than intrinsic strain limitations. Future studies should examine the stability of PGP characteristics in lyophilized *K. radicincitans* after an extended period of storage and colonization dynamics using CFU counts and strain-specific qPCR. Additionally, fluorescent or luminescent reporter strains may allow real-time visualization of spatial distribution and activity within root tissues. Fluorescent reporter strains could further allow real-time visualization of colonization patterns. These approaches would help relate colonization patterns to the observed biological effects.

Detached-leaf assays, while widely used, disrupt vascular continuity and preclude assessment of active systemic signaling (Martínez-Medina et al., 2013; Martínez-Hidalgo et al., 2015). Whole-plant infection assays would strengthen conclusions on systemic resistance but were not conducted due to technical constraints. Future work should combine whole-plant and detached-leaf assays.

Simultaneously investigating two contrasting pathosystems limited mechanistic depth. The necrotrophic pathogen *B. cinerea* is predominantly counteracted by jasmonic acid/ethylene (JA/ET)-mediated defenses, whereas resistance against the hemibiotrophic pathogen *F. oxysporum* is largely associated with salicylic acid (SA)-dependent responses (Glazebrook, 2005; AbuQamar et al., 2008; Di et al., 2017). Crosstalk between SA and JA signaling is highly dynamic and often antagonistic, making conclusions necessarily preliminary (Pieterse et al., 2012; Aerts et al., 2021). Future studies should investigate each pathosystem independently using dedicated mechanistic approaches.

Gene expression was analyzed at a single time point (72 h post-challenge), which reflects the biologically relevant stage of lesion establishment for *B. cinerea* in detached-leaf assays; however, this precludes the temporal resolution required to formally demonstrate priming. Future studies should include time-course analyses (e.g., 6, 12, 24, and 48 h post-inoculation) to determine whether *P. phytofirmans* pre-inoculation accelerates or amplifies defense gene induction compared to non-bacterized controls. This should be complemented by biochemical readouts including quantification of endogenous SA and JA by liquid chromatography–mass spectrometry, measurement of reactive oxygen species accumulation, and assays for phenylalanine ammonia-lyase and PR proteins. Functional validation using hormone-signaling mutants such as SA-deficient *NahG* or *sid2* lines would further clarify the signaling pathway dependency of the resistance phenotype observed here (Thaler et al., 2012; Pieterse et al., 2014).

### 4.5 Conclusion

The two PGPB strains differed markedly in their ability to combat fungal tomato diseases. Although both showed similar antagonistic effects against *B. cinerea* mycelium, necrotic lesions caused by the foliar pathogen *B. cinerea were smaller* in *P. phytofirmans*-inoculated plants. *Paraburkholderia phytofirmans* promoted plant growth, while *K. radicincitans* did not. Most SA-, JA-, and ET-pathway marker genes were unaffected by bacterial treatment. SlPR1a, however, was only induced when *P. phytofirmans*-colonized plants were challenged with the pathogen consistent with a primed defense state. Overall, the results support a priming-associated or ISR-like response in which root inoculation with *P. phytofirmans* is linked to enhanced resistance in distal tomato leaf tissue against *B. cinerea* under detached-leaf conditions; however, definitive mechanistic confirmation remains lacking. Further investigation using time-course gene expression analyses and whole-plant infection assays is needed to clarify the underlying mechanism. Neither strain provided protection against *F. oxysporum* in greenhouse conditions despite *in vitro* antagonism. This underscores that *in vitro* antagonism is an unreliable predictor of *in planta* biocontrol efficacy.

## Data Availability

The datasets generated and/or analyzed during this study are included in the article, and further inquiries can be directed to the corresponding author.
