# Supplementary material for: Biocontrol of Kosakonia radicincitans and Paraburkholderia phytofirmans against Botrytis and Fusarium in tomato: role of potential inducing resistance and pathogen-specific responses
Source: Front Plant Sci. 2026 May 14;17:1775113. doi: 10.3389/fpls.2026.1775113 (PMC13216485; doi:10.3389/fpls.2026.1775113)
Supplement: Supplementary file 1 [file DataSheet1.docx]

Supplementary Material

**Table S1** Oligonucleotide sequences used for the detection and quantification of bacteria in the roots and shoots.

| **Primer name** | **Target protein** |  | **Oligonucleotide sequence (5'-3')** | **Tm** | **Amplicon size (bp)** | **Reference** |
| --- | --- | --- | --- | --- | --- | --- |
| FdnaJ | Chaperone protein | For | AAGCCAGCGTTCCGTCGTA | 72 | 140 | (Witzel et al., 2017) |
|  |  | Rev | GATCGTTGAACTCGTCGAGCAG |  |  |  |
| Bphyt_1824 | Transcription termination factor Rho | For | AAAAACGAGCCAAAACGGGC | 59 | 207 | (Sheibani-Tezerji et al., 2015) |
|  |  | Rev | CACCAGCGCGAAATAACG |  |  |  |
|  |  | Probe | 6-FAM- AAACCTCGTA CCTCGCCAGC-BHQ-1 |  |  |  |

**Table S2** List of primers used for housekeeping plant genes.

| **Primer name** | **Target protein** |  | **Oligonucleotide sequence (5'-3')** | **Tm** | **Amplicon size (bp)** | **Reference** | **NCBI Accession** |
| --- | --- | --- | --- | --- | --- | --- | --- |
| *SlUbi3* | Ubiquitin | For Rev | TCGTAAGGAGTGCCCTAATGCTGA CAATCGCCTCCAGCCTTGTTGTAA | 60 | 119 | (Mascia et al., 2010) | X58253 |
| *SIEF α* | Elongation  factor 1α | For  Rev | GATTGGTGGTATTGGAACTGTC  AGCTTCGTGGTGCATCTC | 55 | NA | (Rotenberg et al., 2006) | X14449 |
| *SlACT* | Actin | For  Rev | GAAATAGCATAAGATGGCAGACG  ATACCCACCATCACACCAGTAT | 60 | 60 | (Løvdal and Lillo, 2009) | BT012695 |
| *SlGAPDH* | glyceraldehyde-3-phosphate dehydrogenase | For  Rev | ACCACAAATTGCCTTGCTCCCTTG  ATCAACGGTCTTCTGAGTGGCTGT | 60 | 60 | (Mascia et al., 2010) | U93208 |
| *SlTUB* | Tubulin | For  Rev | CAAGAACTCGTCCTACTTTG  GCTCACTCACCCTTCTAA | 60 | 146 | (Abbasi et al., 2019) | NA |
| *SlTUB* *α* | α-Tubulin | For  Rev | TCGTGGCCACTATACCATTG  AGTGACCCAAGACCTGAACC | 60 | NA | (Di et al., 2017) | NA |

NA: Data are not available in the reference.

**Table S3** List of primers used in the expression analysis of plant defence-related genes.

| **Primer name** | **Target protein** |  | **Oligonucleotide sequence (5'-3')** | **Tm** | **Amplicon size (bp)** | **Reference** | **NCBI Accession** |
| --- | --- | --- | --- | --- | --- | --- | --- |
| *SlPR1a* | Pathogenesis-related protein | For  Rev | GTGGGATCGGATTGATATCCT  CCTAAGCCACGATACCATGAA | 60 | NA | (Martínez-Medina et al., 2013) | M69247 |
| *SlWRKY70* | WRKY transcription factor 70 | For  Rev | TGGTAAAGCATAGTGACTCAAC  AGAGGGAGAAGAAGGCATAA | 60 | 131 | (Abbasi et al., 2019) | NA |
| *SlPin II* | Proteinase inhibitor II | For  Rev | GAAAATCGTTAATTTATCCCAC  ACATACAAACTTTCCATCTTTA | 60 | NA | (Uppalapati et al., 2005) | K03291 |
| *SlLoxA* | Lipoxygenase A | For  Rev | GGTTACCTCCCAAATCGTCC  TGTTTGTAACTGCGCTGTG | 60 | NA | (López-Ráez et al., 2010) | U09026 |
| *SlDEF4* | Defensin-like proteins 4 | For  Rev | AAAAAGTGGCAAGTGGAATGG  AATGGCAAGGTGAGTAGCAGTAA | 60 | NA | (Nikoloudakis et al., 2020) | XM_004242790 |
| *SlERF1* | Ethylene response factor 1 | For  Rev | AGACTTGGGAGTTGAATTA  TACATTGCGATCTTGATTA | 60 | 126 | (Abbasi et al., 2019) | NA |

NA: Data are not available in the reference.

**Table S4** Biocontrol efficacy of two plant growth-promoting bacteria, *Kosakonia radicincitans* and *Paraburkholderia phytofirmans* against grey mould of tomato caused by *Botrytis cinerea* was calculated. Values are the means averaged ± SD from five replicates of the bioassay. The same letters within each column represent a non-significant difference of 5%, according to Bonferroni correction for multiple comparisons after conducting the Kruskal-Wallis test (*p* < 0.05, *n* = 10).

| **Treatment** | **Biocontrol efficacy (%)** |
| --- | --- |
| Control | 0^a^ |
| *K. radicincitans* | 5.52 ± 22.12^ab^ |
| *P. phytofirmans* | 19.55 ± 29.07^b^ |

**Table S5** Disease severity of *Fusarium oxysporum* FOL and biocontrol efficacy of two plant growth-promoting bacteria, *Kosakonia radicincitans* and *Paraburkholderia phytofirmans* against wilt tomato disease caused by FOL were calculated. The same letters within each column represent a non-significant difference of 5%, according to Bonferroni correction for multiple comparisons after conducting the Kruskal-Wallis test (*p* < 0.05, *n* = 4).

| **Treatment** | **Disease severity** | **Biocontrol efficacy [%]** |
| --- | --- | --- |
| Control | 4.25^a^ | 0^a^ |
| *K. radicincitans* | 4.25^a^ | 0^a^ |
| *P. phytofirmans* | 4^a^ | 5^a^ |


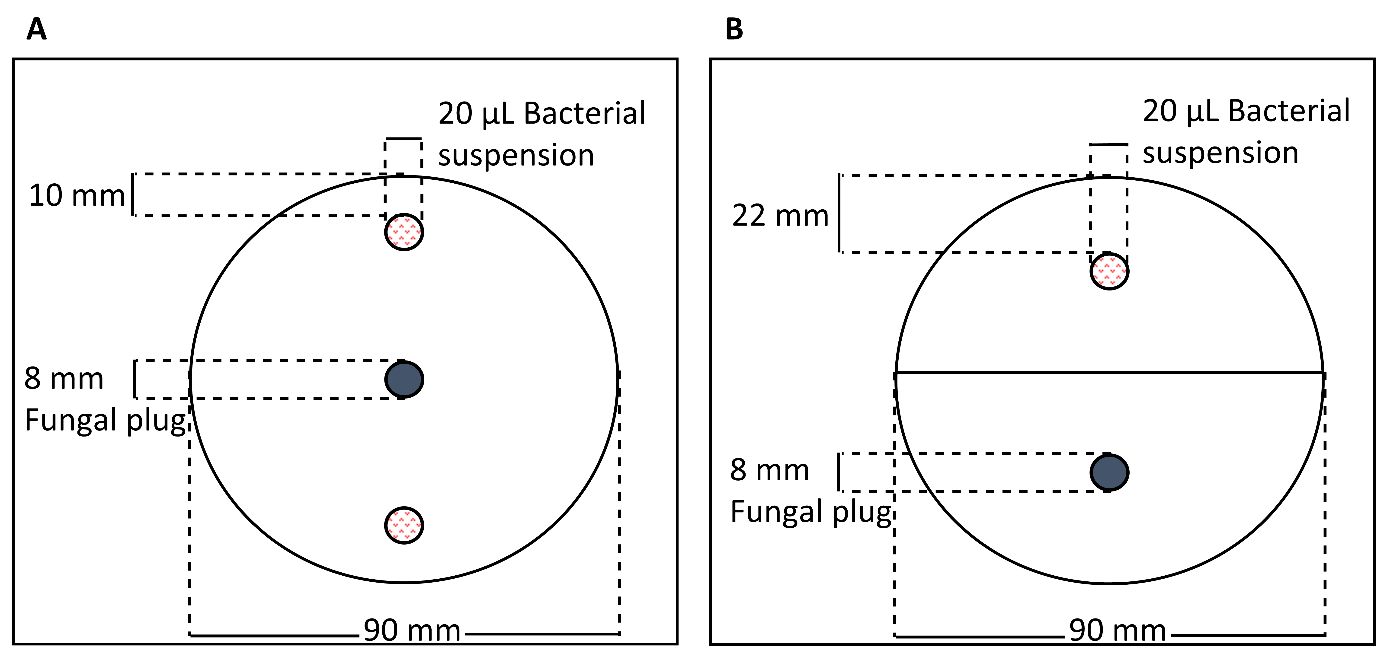


**Figure S1**

Schematic diagram of the *in vitro* antagonistic assay against *Botrytis cinerea* or *Fusarium oxysporum* f.sp. *lycopersici*. (**A**) Dual culture: a 20 µL-bacterial suspension of *Kosakonia radicincitans* or *Paraburkholderia phytofirmans* with a concentration of 10^8^ CFU mL^-1^ or mock (10 mM MgCl_2_ buffer) was grown on Potato dextrose agar (PDA) medium plates at the two opposite sides (1 cm from the plate edge) for two days at 24°C in darkness in the dual culture before an 8-mm diameter plug of fungal mycelium was placed in the middle of the plate for 10 days for *B. cinerea* and 4 days for *F. oxysporum*. (**B**) Two-compartment plates: a 20 µL-bacterial suspension of *K. radicincitans* or *P. phytofirmans* with 10^8^ CFU mL^-1^ or mock (10 mM MgCl_2_ buffer) was grown on PDA medium plates in the middle of one-half of the plates. After two days, a plug of fungi was placed in the other side of the two-compartment Petri dish for 10 and 4 days with *B. cinerea* or *F. oxysporum*, respectively, at 24°C in darkness.


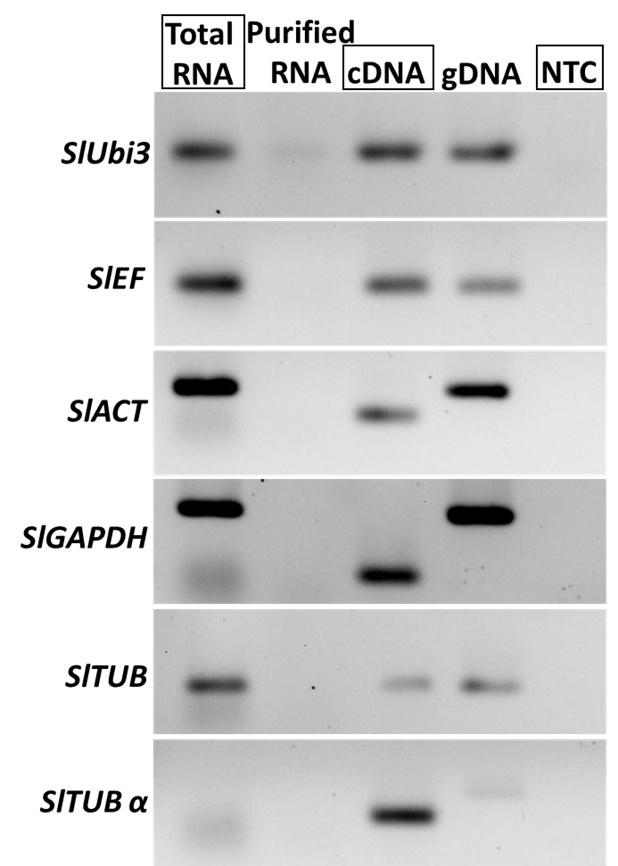


**Figure S2**

Agarose gel electrophoresis of PCR products of specific primer pairs for six reference genes of samples of tomato leaf extracted total RNA, purified RNA, cDNA, genomic DNA, and no template control (NTC) obtained with the specific primer pairs for the tomato housekeeping genes encoding ubiquitin 3 (*Ubi3*), elongation factor 1α (*SlEF*), actin (*SlACT*), glyceraldehyde 3-phosphate dehydrogenase (*SlGAPDH*), tubulin (*SlTUB*) and α-tubulin (*SlTUB α*). Wells were loaded with 10 µL of PCR product per gel pocket after mixing with the loading dye. Electrophoresis was conducted in a 1% agarose gel (w/v) containing 0.25 µg µL^-1^ of ethidium bromide in 1x Tris-acetate-EDTA (TAE) buffer at 80 V for 1 hour.


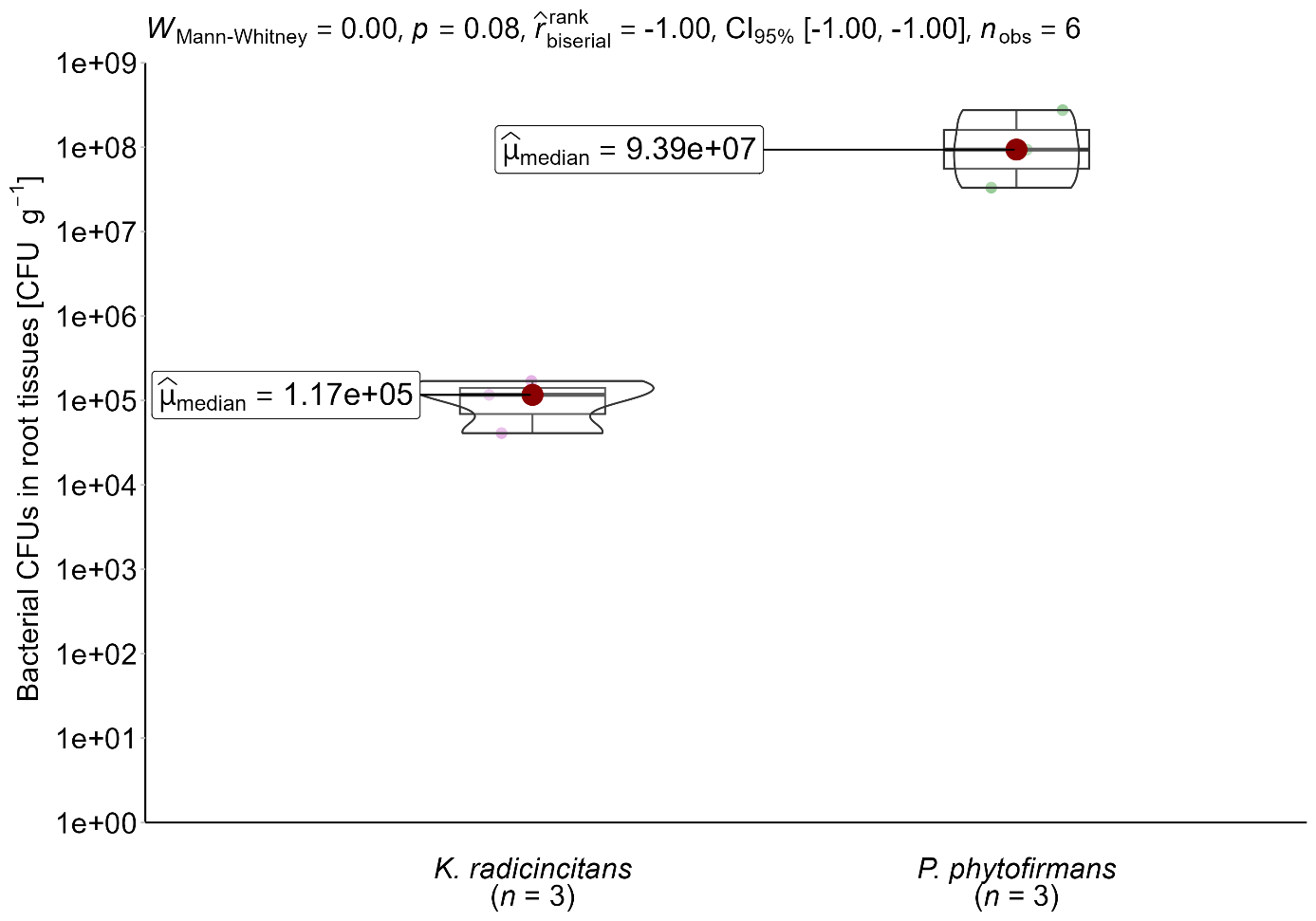


**Figure S3**

Quantification of bacteria in colonized roots via qPCR after one month of inoculation. Values represent bacterial colony format units (CFU) per gram (g) of fresh root weight based on the absolute quantification of the standard curve of a ten-fold serial dilution series for *Kosakonia radicincitans* or *Paraburkholderia phytofirmans*. Data are presented as box/violin plots showing the distribution of the data in the inner box with the median (black line) and the interquartile range. Furthermore, the average of the median ("µ") is displayed, and the circled dots indicate the values for each bacterial treatment. A rank bi-serial correlation coefficient with 95% confidence intervals is shown as a measure of effect size. A Mann–Whitney test with a Wilcoxon rank sum test with continuity correction was used to analyse differences between the two treatments (*p* = 0.08). Total replicates (*n* = 3) comprised three biological replicates, each including three technical replicates.


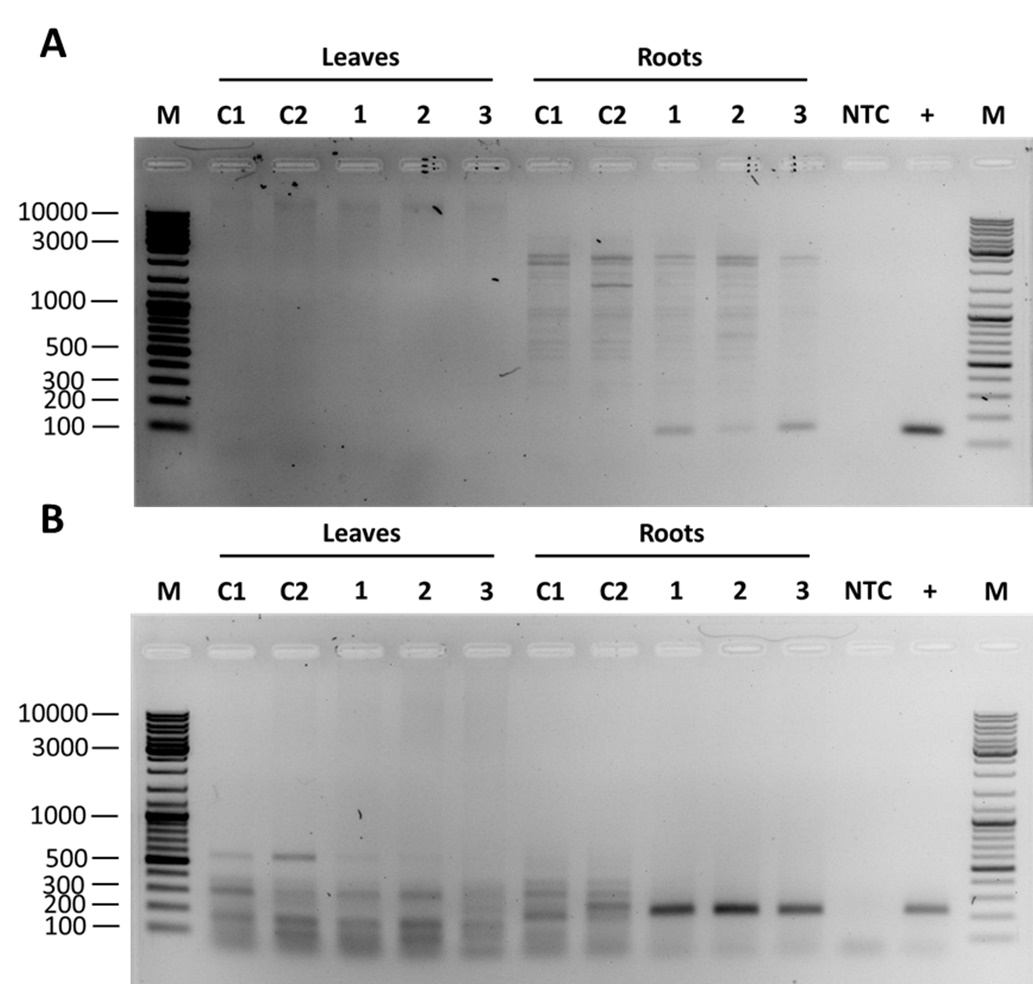


**Figure S4**

Agarose gel electrophoresis of qPCR products of (**A**) the *Kosakonia radicincitans* gene *FdnaJ* and (**B**) the *Paraburkholderia phytofirmans* gene *phyt_1824*. PCR products from DNA samples of leaves and roots of plants inoculated at transplanting with bacterial inocula and used for bio-protection and gene expression analysis are shown in lanes 1, 2, and 3, while PCR products from DNA samples of non-inoculated plants are shown in lane C1 and C2 as negative controls. Pure genomic DNA of bacteria (10^8^ CFU mL^-1^) was used as template for the positive control (+), while the respective no template control is shown in lane NTC. The sizes of the molecular weight markers (M) were given on the left and right in base pairs.

References

Abbasi, S., Safaie, N., Sadeghi, A., and Shamsbakhsh, M. (2019). *Streptomyces* strains induce resistance to *Fusarium oxysporum* f. sp. *lycopersici* race 3 in tomato through different molecular mechanisms. *Front. Microbiol.* 10, 1505. doi: 10.3389/fmicb.2019.01505

Di, X., Gomila, J., and Takken, F. L. W. (2017). Involvement of salicylic acid, ethylene and jasmonic acid signalling pathways in the susceptibility of tomato to *Fusarium oxysporum*. *Mol. Plant Pathol.* 18, 1024–1035. doi: 10.1111/mpp.12559

López-Ráez, J. A., Verhage, A., Fernández, I., García, J. M., Azcón-Aguilar, C., Flors, V., et al. (2010). Hormonal and transcriptional profiles highlight common and differential host responses to arbuscular mycorrhizal fungi and the regulation of the oxylipin pathway. *J. Exp. Bot.* 61, 2589–2601. doi: 10.1093/jxb/erq089

Løvdal, T., and Lillo, C. (2009). Reference gene selection for quantitative real-time PCR normalization in tomato subjected to nitrogen, cold, and light stress. *Anal. Biochem.* 387, 238–242. doi: 10.1016/j.ab.2009.01.024

Martínez-Medina, A., Fernández, I., Sánchez-Guzmán, M. J., Jung, S. C., Pascual, J. A., and Pozo, M. J. (2013). Deciphering the hormonal signalling network behind the systemic resistance induced by *Trichoderma harzianum* in tomato. *Front. Plant Sci.* 4, 206. doi: 10.3389/fpls.2013.00206

Mascia, T., Santovito, E., Gallitelli, D., and Cillo, F. (2010). Evaluation of reference genes for quantitative reverse-transcription polymerase chain reaction normalization in infected tomato plants. *Mol. Plant Pathol.* 11, 805–816. doi: 10.1111/j.1364-3703.2010.00646.x

Nikoloudakis, N., Pappi, P., Markakis, E. A., Charova, S. N., Fanourakis, D., Paschalidis, K., et al. (2020). Structural diversity and highly specific host-pathogen transcriptional regulation of defensin genes is revealed in tomato. *Int. J. Mol. Sci.* 21. doi: 10.3390/ijms21249380

Rotenberg, D., Thompson, T. S., German, T. L., and Willis, D. K. (2006). Methods for effective real-time RT-PCR analysis of virus-induced gene silencing. *J. Virol. Methods* 138, 49–59. doi: 10.1016/j.jviromet.2006.07.017

Sheibani-Tezerji, R., Rattei, T., Sessitsch, A., Trognitz, F., and Mitter, B. (2015). Transcriptome profiling of the endophyte *Burkholderia phytofirmans* PsJN indicates sensing of the plant environment and drought stress. *mBio* 6, e00621-15. doi: 10.1128/mBio.00621-15

Uppalapati, S. R., Ayoubi, P., Weng, H., Palmer, D. A., Mitchell, R. E., Jones, W., et al. (2005). The phytotoxin coronatine and methyl jasmonate impact multiple phytohormone pathways in tomato. *TPJ* 42, 201–217. doi: 10.1111/j.1365-313X.2005.02366.x

Witzel, K., Strehmel, N., Baldermann, S., Neugart, S., Becker, Y., Becker, M., et al. (2017). *Arabidopsis thaliana* root and root exudate metabolism is altered by the growth-promoting bacterium *Kosakonia radicincitans* DSM 16656^T^. *Plant Soil* 419, 1–17. doi: 10.1007/s11104-017-3371-1
